# Frequency Preference Response to Oscillatory Inputs in Two-dimensional Neural Models: A Geometric Approach to Subthreshold Amplitude and Phase Resonance

**DOI:** 10.1186/2190-8567-4-11

**Published:** 2014-05-08

**Authors:** Horacio G Rotstein

**Affiliations:** 1 Department of Mathematical Sciences, New Jersey Institute of Technology, Newark, NJ, 07102, USA

## Abstract

We investigate the dynamic mechanisms of generation of subthreshold and phase resonance in two-dimensional linear and linearized biophysical (conductance-based) models, and we extend our analysis to account for the effect of simple, but not necessarily weak, types of nonlinearities. Subthreshold resonance refers to the ability of neurons to exhibit a peak in their voltage amplitude response to oscillatory input currents at a preferred non-zero (resonant) frequency. Phase-resonance refers to the ability of neurons to exhibit a zero-phase (or zero-phase-shift) response to oscillatory input currents at a non-zero (phase-resonant) frequency. We adapt the classical phase-plane analysis approach to account for the dynamic effects of oscillatory inputs and develop a tool, the envelope-plane diagrams, that captures the role that conductances and time scales play in amplifying the voltage response at the resonant frequency band as compared to smaller and larger frequencies. We use envelope-plane diagrams in our analysis. We explain why the resonance phenomena do not necessarily arise from the presence of imaginary eigenvalues at rest, but rather they emerge from the interplay of the intrinsic and input time scales. We further explain why an increase in the time-scale separation causes an amplification of the voltage response in addition to shifting the resonant and phase-resonant frequencies. This is of fundamental importance for neural models since neurons typically exhibit a strong separation of time scales. We extend this approach to explain the effects of nonlinearities on both resonance and phase-resonance. We demonstrate that nonlinearities in the voltage equation cause amplifications of the voltage response and shifts in the resonant and phase-resonant frequencies that are not predicted by the corresponding linearized model. The differences between the nonlinear response and the linear prediction increase with increasing levels of the time scale separation between the voltage and the gating variable, and they almost disappear when both equations evolve at comparable rates. In contrast, voltage responses are almost insensitive to nonlinearities located in the gating variable equation. The method we develop provides a framework for the investigation of the preferred frequency responses in three-dimensional and nonlinear neuronal models as well as simple models of coupled neurons.

## 1 Introduction

Rhythmic oscillations have been observed in various areas of the brain and have been implicated in cognition and motor behavior [1-4] in both health and disease [5]. Network oscillations result from the cooperative activity of the participating neurons [3]. Many neuron types possess membrane potential oscillatory properties [4], which emerge either in the form of intrinsic subthreshold oscillations (STOs) [4,6-9], or subthreshold resonance [10-20], or both [12,15,21,22]. Subthreshold resonance refers to the ability of neurons to exhibit a peak in their voltage amplitude response to oscillatory input currents at a preferred non-zero (resonant) frequency [10]. Intrinsic STOs in isolated neurons emerge spontaneously or in response to tonic (DC) current inputs, and primarily reflect interactions among the neuron’s intrinsic currents. In contrast, subthreshold resonance results from the interaction between these intrinsic currents and an oscillatory input currents. Because of this, subthreshold resonance has been implicated in the generation of oscillations at the network level [23-27]. 

The relation between intrinsic STOs and subthreshold resonance is still an open question. For some neuron types, STOs and subthreshold resonance have been shown to result from the same mechanism [12]. However, theoretical and experimental studies have demonstrated that they are not equivalent phenomena [11,28]: neurons may exhibit one and not the other [10,11,21]. Furthermore, standard calculations for linear models show that their natural (intrinsic) and resonant frequencies do not generally coincide except in some, rather restricted, parameter regimes [11,29] (see also our discussion in Appendix A.3).

The phase-shift (or phase) of the neuronal voltage response to subthreshold oscillatory input currents has received less attention that the corresponding amplitude response [11,14,29]. This despite the fact that phases are expected to play a major role in determining the synchronization properties of neuronal networks [30]. A zero-phase response indicates that both voltage output and current input peak at the same time, thus generating in-phase synchronized patterns. We use the term phase-resonance to refer to the ability of neurons to exhibit a zero-phase response to oscillatory inputs at a non-zero (phase-resonant) frequency. The resonant and phase-resonant frequencies do not generally coincide [29] for neuronal models (they do so for circuits in parallel but not for circuits in series as neuronal models are). In addition, resonance may occur in the absence of phase-resonance [29] (see also our discussion in Appendix A.3).

The properties of subthreshold resonance have been investigated in many systems [9,11,14,15,20,21,25,29]. Theoretical studies have focused on simulations of conductance-based models and the analysis of the impedance profiles (curves of amplitude and phase-shift as a function of the input frequency) for the corresponding linearized models around resting potential [11,14-16,18,31-35]. However, the mechanisms underlying the generation of resonance and phase-resonance in neurons are not fully understood. This is partly because adequate tools are lacking. 

These mechanisms can be addressed from two different, but complementary perspectives: biophysical and dynamic. The former focuses on the role of the ionic currents and their biophysical properties in shaping the neuron’s voltage response, and it has been discussed in terms of the so-called resonant and amplifying ionic currents (see Sect. 2.5) [10,11]. The latter focuses on (i) the geometric properties of the neuronal models in terms of the nullclines and phase planes, and (ii) the interaction between the neuron’s intrinsic time scales and the time scales associated with the input currents to produce optimal voltage responses in both amplitude and phase. 

In [29] we have identified the basic biophysical mechanisms of generation of resonance and phase-resonance in two-dimensional linear and linearized conductance-based models, and we have conducted a thorough study of the properties of the voltage response in terms of the biophysical parameters. In particular, we have shown how changes in the maximal conductances affect the resonant and phase-resonant frequencies and other attributes of the impedance amplitude and phase profiles in ways that are not always intuitive. 

The goal of this paper is to investigate the dynamic mechanisms that give rise to resonance and phase-resonance. Specifically, we aim to identify the basic dynamic and geometric principles that govern the generation of these phenomena in order to understand (i) how the resonant and phase-resonant frequencies are selected, (ii) how the voltage response is amplified at the resonant frequency band, (iii) how these properties are affected by changes in parameters (e.g., maximal conductances, time constants), and (iv) how resonance and phase-resonance are related to intrinsic STOs.

We use dynamical systems tools and numerical simulations, and we extend the classical phase-plane analysis approach to generate a tool (envelope-plane diagrams) that enables us to address the mechanistic issues described above. For simplicity, in this paper we focus on two-dimensional linear systems and we illustrate how the ideas we develop here can be used to investigate linearized conductance-based models and nonlinear models.

From a dynamic perspective, we view the properties of the forced oscillations as reflecting the interaction between the forced neuron’s intrinsic time scales, which result from a combination of its biophysical properties and the time scales of the input. The time scales that emerge from these interactions determine the amplitude and phase of the voltage response for each value of the input frequency. The resonant and phase-resonant frequencies correspond to the emergent time scales that allow the neuron to maximize its voltage amplitude response and to peak in phase with the input, respectively. Therefore, we aim to elucidate how these emergent time scales are generated, how intrinsic and emergent time scales are related, and how they are affected by changes in the model parameter.

It is clear that for linear systems the voltage response can be computed analytically. However, due to its complexity, the dependence of the voltage response properties with the model parameters is not straightforward, not even for linear two-dimensional systems [29]. Additionally, due to the same complexity, it is difficult to extract from the closed-form solutions a mechanistic understanding of the neuron’s resonant properties in terms of the time scales. The use of geometric tools aids in this effort. Since the phase plane contains information about the biophysical properties of neurons through the structure of the nullclines and time scales, dynamical systems tools allow us to make statements that are valid for generic classes of biophysical models. 

## 2 Methods

### 2.1 Conductance-Based Models

We consider conductance-based models of Hodgkin–Huxley type [36]. The current-balance equation is given by

(1) $$C\frac{dV}{dt}=-I_{L}-I_{1}-I_{2}+I_{\mathrm{app}}+I_{\mathrm{in}}(t),$$

where *V* is the membrane potential (mV), *t* is time measured in msec, *C* is the membrane capacitance (μF/cm^2^), $I_{\mathrm{app}}$ is the applied bias (DC) current (μA/cm^2^), $I_{\mathrm{in}}(t)$ is a time-dependent input current (μA/cm^2^), $I_{L}=G_{L}(V-E_{L})$ is the leak current, and $I_{j}$ (j=1,2) are ionic currents of the form

(2) $$I_{j}=G_{j}x_{j}(V-E_{j})$$

with maximal conductance $G_{j}$ (mS/cm^2^) and reversal potentials $E_{j}$ (mV), respectively. The ionic currents (2) we consider here are restricted to have a single gating variable $x_{j}$ and to be linear in $x_{j}$. This choice is motivated by the persistent sodium ($I_{\mathrm{Nap}}$), h- ($I_{h}$) and M-type ($I_{M}$) currents found in several neurons that exhibit subthreshold resonance [15,37-39] (see Appendix B). All gating variables *x* obey a first order differential equation of the form

(3) $$\frac{dx}{dt}=\frac{x_{\infty }(V)-x}{\tau_{x}(V)},$$

where $x_{\infty }(V)$ and $\tau_{x}(V)$ are the voltage-dependent activation/inactivation curves and time scales, respectively. For external sinusoidal inputs we use the following notation:

(4) $$I_{\mathrm{in}}(t)=A_{\mathrm{in}}\sin(\Omega t)\;\text{with }\Omega =\frac{2\pi f}{T},$$

where T=1000 msec and [f]= Hz.

In this paper we focus on two-dimensional models having one dynamic gating variable ($x_{1}$) and, possibly, an additional gating variable evolving on a fast time scale for which the adiabatic approximation $x_{2}=x_{2,\infty }(V)$ is made. Additional fast currents can be including without significantly changing the formalism. The investigation of three-dimensional systems is beyond the scope of this paper.

### 2.2 Linearized Conductance-Based Models

We follow Richardson et al. [11] and linearize the autonomous part of system (1)–(3) ($I_{\mathrm{in}}=0$) around the fixed point $\left(\bar{V},{\bar{x}}_{1}\right)$ by defining

(5) $$v=V-\bar{V}\;\text{and}\;w=\frac{x_{1}-{\bar{x}}_{1}}{x_{1,\infty }^{\prime }(\bar{V})},$$

where ${\bar{x}}_{1}=x_{1,\infty }(\bar{V})$. The linearized equations are [11]

(6) $$C\frac{dv}{dt}=-g_{L}v-g_{1}w+I_{\mathrm{in}}(t),$$

(7) $${\bar{\tau }}_{1}\frac{dw}{dt}=v-w,$$

where the effective conductances $g_{L}$ and $g_{1}$, and time constant ${\bar{\tau }}_{1}$ are defined by

(8) $$g_{L}=G_{L}+G_{1}x_{1,\infty }(\bar{V})+G_{2}x_{2,\infty }(\bar{V})+g_{2},$$

(9) $$g_{j}=G_{j}(\bar{V}-E_{j})x_{j,\infty }^{\prime }(\bar{V}),\;j=1,2$$

and

(10) $${\bar{\tau }}_{1}=\tau_{1}(\bar{V}).$$

The sign of the effective ionic conductances $g_{j}$ (j=1,2) determines whether their associated gating variables are resonant ($g_{j}>0$) or amplifying ($g_{j}<0$) [10,11]. The effective conductance $g_{L}$ includes information not only about the original leak conductance $G_{L}$ but also about the voltage-dependent (“passive”) terms of the ionic currents. The ionic currents $I_{1}$ and $I_{2}$ each contribute a positive term to $g_{L}$. The ionic current $I_{2}$ contributes to $g_{L}$ with an additional term ($g_{2}$) due to its instantaneous dynamics. If $x_{2}$ is amplifying and $g_{2}<0$ is large enough in absolute value, then $g_{L}$ is negative. Note that the gating variable *w* in (5) has units of voltage.

### 2.3 Rescaled Linearized Models

We rescale system (6)–(7) to aid in the analysis and to reduce the number of parameters that effectively govern its dynamics (without loss of information). The rescaling we use here focuses on the geometric properties of the system and the time-scale separation between the participating variables, and is amenable to analysis using dynamical systems tools (phase-plane analysis). A different scaling, appropriate for addressing biophysically related questions, has been used in [11,29]. 

We define the following dimensionless time and (dimensional) voltage variables:

(11) $$\hat{t}=\frac{g_{L}}{C}t,\;\;\hat{v}=\frac{g_{L}}{g_{1}}v=\frac{v}{\alpha },$$

parameters

(12) $$\alpha =\frac{g_{1}}{g_{L}},\;\;\epsilon =\frac{C}{{\bar{\tau }}_{1}g_{L}},$$

and

(13) $$\begin{array}{rl} {\hat{I}}_{\mathrm{in}}(t) & ={\hat{A}}_{\mathrm{in}}\sin(2\pi f\hat{t}/\hat{T})\;\text{with}\\ {\hat{A}}_{\mathrm{in}} & =\frac{A_{\mathrm{in}}}{g_{1}},\;\;\hat{T}=\frac{Tg_{L}}{C}\;\text{and}\;\hat{\Omega }=\frac{2\pi f}{\hat{T}}. \end{array}$$

Substituting into (6)–(7) and dropping the “hat” signs we obtain

(14) $$\frac{dv}{dt}=-v-w+A_{\mathrm{in}}\sin(\Omega t),$$

(15) $$\frac{dw}{dt}=\epsilon [\alpha v-w].$$

Geometrically, the parameter *α* is the slope of the *w*-nullcline and can be thought of as representing the strength of the gain of the feedback in the linearized system. The parameter *ϵ* represents the time-scale separation between *v* and *w*.

**Fig. 1:**
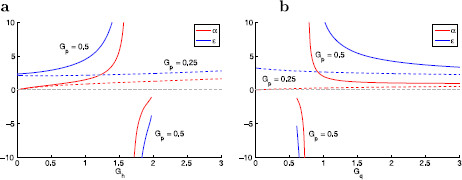
Dimensionless parameters *α* and *ϵ* as a function of the biophysical resonant conductances in two representative conductance-based models. The models are described in Appendix B. **a**$I_{h}+I_{\mathrm{Nap}}$ model with slow $I_{h}$ and fast $I_{\mathrm{Nap}}$ (biophysical conductances: $G_{h}$ and $G_{p}$, respectively). **b**$I_{\mathrm{Ks}}+I_{\mathrm{Nap}}$ model with slow $I_{\mathrm{Ks}}$ and fast $I_{\mathrm{Nap}}$ (biophysical conductances: $G_{q}$ and $G_{p}$, respectively). For small enough values of $G_{p}$ (*dashed curves*) both *α* and *ϵ* are positive for all values of $G_{h}$ and $G_{q}$ (**a** and **b**, respectively), while for larger values of $G_{p}$ (*solid curves*) both *α* and *ϵ* are negative for large values of $G_{h}$ and small values of $G_{q}$ (in **a** and **b**, respectively). Note that although $I_{h}$ and $I_{\mathrm{Ks}}$ are resonant currents, both *α* and *ϵ* exhibit different monotonic properties as $G_{h}$ (**a**) and $G_{q}$ (**b**) increase

Since for resonant gating variables $g_{1}>0$, the sign of both *α* and *ϵ* depends on whether $g_{L}$ is positive or negative. In the absence of fast amplifying currents ($G_{2}=g_{2}=0$), $g_{L}>0$ and then both α>0 and ϵ>0. When an amplifying current is present and its contribution to $g_{L}$ is small enough, the sign of both *α* and *ϵ* remains positive (Fig. 1, dashed curves). However, when stronger contributions of the fast amplifying current causes $g_{L}$ to be negative, the sign of both *α* and *ϵ* are also negative (Fig. 1, solid curves). Resonance occurs in both cases [11,29]. Since resonance becomes amplified as $g_{L}$ decreases [29], we expect resonance to be more amplified for negative values of both *ϵ* and *α* as compared to positive ones. The cases including values of *α* and *ϵ* having different signs are excluded from this study since the underlying autonomous system is either unstable (saddle) or stable (node) but does not exhibit resonance (Fig. 3). In both cases $x_{1}$ is amplifying ($g_{1}<0$). The case ϵ<0 and α>0 requires that both $g_{L}<0$ and $g_{1}<0$, while the case ϵ>0 and α<0 requires that $g_{L}>0$ and $g_{1}<0$. 

### 2.4 Impedance and Impedance-Like Functions

The voltage response of a linear system receiving sinusoidal current inputs of the form (4) is given by

(16) $$V_{\mathrm{out}}(t;f)=A_{\mathrm{out}}(f)\sin\left(\Omega t-\phi (f)\right),$$

where $A_{\mathrm{out}}$ is the amplitude and *ϕ* is the phase-shift (or phase), defined as the difference between the peaks of the current input $I_{\mathrm{in}}(t;f)$ and the voltage output $V_{\mathrm{out}}(t;f)$.

**Fig. 2:**
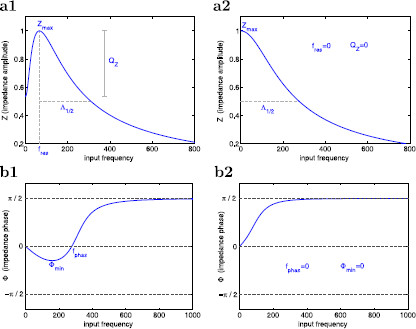
Schematic diagrams of the impedance (**a**) and phase (**b**) profiles (impedance and phase as a function of the input frequency *f*). **a1** Band-pass filter (resonance). **a2** Low-pass filter (no resonance). **b1** Zero-frequency phase crossing (phase-resonance). **b2** Monotonically increasing and positive phase (no phase-resonance). **a** The resonant frequency $f_{\mathrm{res}}$ is the input frequency *f* at which the impedance Z(f) reaches its maximum $Z_{\max}$. The resonance amplitude $Q_{Z}=Z_{\max}-Z(0)$ measures the resonance power. The half-width frequency band $\Lambda_{1/2}$ is the length of the frequency interval in between $f_{\mathrm{res}}$ and the input frequency value at which $Z(f)=Z_{\max}/2$, and measures the system’s selectivity to incoming frequencies close to $f_{\mathrm{res}}$. **b** The phase-resonant frequency $f_{\mathrm{phas}}$ is the zero-crossing phase frequency. The minimum phase $\phi_{\min}$ measures the magnitude of the negative phase

Linear systems exhibit resonance if there is a peak in the amplitude of the impedance function Z(f) given by

(17) $$\left|Z(f)\right|=\frac{A_{\mathrm{out}}(f)}{A_{\mathrm{in}}}$$

at some positive (resonant) frequency $f_{\mathrm{res}}$. In what follows, we will refer to impedance amplitude simply as the impedance Z(f). In Fig. 2a we show representative graphs of the impedance function Z(f) for a model that does (panel a1) and does not (panel a2) exhibit resonance. We characterize the impedance profiles using four parameters: (i) the resonant frequency $f_{\mathrm{res}}$, (ii) the maximum impedance $Z_{\max}=Z(f_{\mathrm{res}})$, (iii) the resonance amplitude $Q_{Z}=Z_{\max}-Z(0)$, and (iv) the half-width frequency band $\Lambda_{1/2}$, defined as the frequency interval in between $f_{\mathrm{res}}$ and the input frequency value at which $Z(f)=Z_{\max}/2$. $\Lambda_{1/2}$ is a measure of the frequency selectivity. Neurons have a higher selectivity to inputs with frequencies around $f_{\mathrm{res}}$ the sharper the graph of Z(f). In Fig. 2b we show two representative graphs of the phase ϕ(f) where *ϕ* vanishes at a non-zero value of *f* (panel b1) and *ϕ* is always positive (panel b2). We refer to the ability of the neuron to exhibit a zero-phase frequency response at a non-zero frequency as *phase-resonance* and to the corresponding frequency as the phase-resonant frequency $f_{\mathrm{phas}}$. In panel b1, $f_{\mathrm{phas}}>0$. The voltage response is “advanced” and “delayed” for lower and higher frequency inputs, respectively. Although phase advance and phase delay are ambiguous concepts to describe phase differences between inputs and outputs in oscillatory systems, we still use them since typical phase differences lie in the range (−π/2,π/2). In panel b2, $f_{\mathrm{phas}}=0$, that is, the voltage response is delayed for all values of *f*. We characterize the phase profiles using two parameters: (i) the phase-resonant frequency $f_{\mathrm{phas}}$, and (ii) the minimum phase $\phi_{\min}$. 

For nonlinear systems, or for linear systems with non-sinusoidal inputs, (17) does no longer provide an appropriate definition of the impedance function. Here we assume that the voltage output is periodic and has the same frequency as the input and we use the following definition:

(18) $$Z(f)=\frac{V_{\max}(f)-V_{\min}(f)}{2A_{\mathrm{in}}},$$

where $V_{\max}(f)$ and $V_{\min}(f)$ are the maximum and minimum of the oscillatory voltage response $V_{\mathrm{out}}(f)$ for each value of the input frequency *f*. For linear systems receiving sinusoidal inputs, (18) and (17) are equivalent. The resonant frequency $f_{\mathrm{res}}$ is the peak frequency of Z(f) in (18). Similarly to the linear case ϕ(f), the phase is computed as the distance between the peaks of the output and input normalized by the period. Note that (18) can be thought of as a filtered version of the impedance function computed using the so-called ZAP functions [10,11,28] that sweep through a given range of frequencies continuously over time. 

### 2.5 Resonant and Amplifying Ionic Currents

Biophysically, subthreshold resonance has been argued to result from a combination of low- and high-pass filter mechanisms that have been described in terms of neural currents [10]. RC circuits act as low-pass filters. (As the input frequency increases the voltage amplitude response of passive neurons decreases from its resistance value, for zero input frequency, to zero, for input frequencies approaching infinity.) 

Ionic currents, or more precisely their associated gating variables, have been classified into resonant ($g_{1}>0$) and amplifying ($g_{1}<0$) [10,11]. Resonant gating variables (e.g., hyperpolarization-activated h-currents [15-17] and slow potassium, M-type currents [14]) have the ability to create resonance by opposing voltage changes (negative feedback). Amplifying gating variables (e.g., persistent sodium currents [14-17] and high-threshold calcium currents [10]) generate a positive feedback effect that enhances voltage changes but they do not create resonance [10]. Some currents such as the low-threshold calcium current $I_{T}$ have both resonant and amplifying gating variables [10]. 

The interaction between resonant and amplifying currents in shaping the neuronal voltage response (impedance and phase profiles) to oscillatory input currents is complex [29], sometimes non-intuitive, and involves not only changes in the maximal amplitude of the voltage response but also in other attributes including the resonant and phase resonant frequencies. The role that different types of resonant and amplifying currents play in shaping the impedance profile has been recently clarified in [29]. An important outcome of this study is that the standard classification “resonant vs. amplifying” does not capture in its entirety the effect that changes in their biophysical parameters have on the shapes of the impedance and phase profiles. For instance, while hyperpolarization-activated (h- or $I_{h}$) and M-type slow potassium ($I_{\mathrm{Ks}}$) currents have qualitatively similar effects on the impedance profile as the corresponding ionic conductances ($G_{h}$ and $G_{\mathrm{Ks}}$), they may have opposite effects in the presence of a persistent sodium current (amplifying). Specifically, in the latter case, an increase in $G_{h}$ leads to an amplification of the voltage response, while an increase in $G_{\mathrm{Ks}}$ causes an attenuation of the voltage response. Additionally, an increase in the time constant associated to the gating variable, which increases the system’s time-scale separation, does not only affect the resonant and phase-resonant frequencies, but it may also produce a significant amplification of the voltage response.

## 3 Results

### 3.1 Stability and Resonant Properties in the *α*–*ϵ* Rescaled System

Here we review the stability and resonant properties of the rescaled equations (14)–(15) in terms of the parameters *α* and *ϵ* for later use. These properties have been discussed in [11,29] for a different rescaling based on dimensionless effective conductances. 

#### 3.1.1 Stability Properties for the Autonomous System and Intrinsic Oscillations

We first consider system (14)–(15) with $A_{\mathrm{in}}=0$. The fixed point is given by $\left(\bar{v},\bar{w})=(0,0\right)$ and the eigenvalues are given by (Appendix A)

(19) $$r_{1,2}=\frac{-(1+\epsilon )\pm \sqrt{{\left(1-\epsilon \right)}^{2}-4\alpha \epsilon }}{2}.$$

**Fig. 3:**
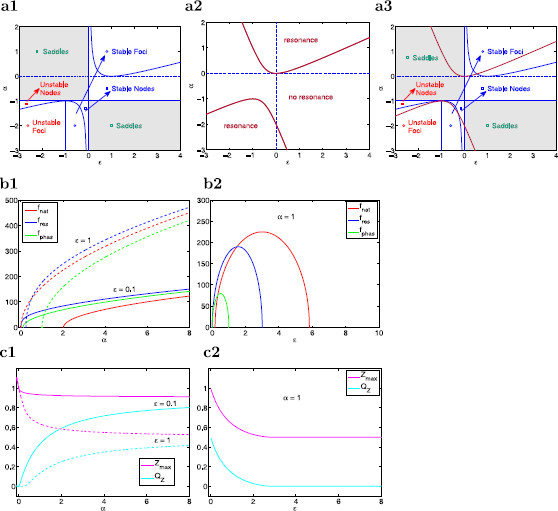
Stability and resonant properties for the reduced two-dimensional linear system (14)–(15). **a** Stability and resonance diagrams in the *α*–*ϵ* parameter space. **a1** Stability diagram for the autonomous system. *The blue curves* separate between regions with different stability properties. **a2** Resonance diagram. *The red curves* separate between regions where the system does (above and below) and does not (middle) exhibit resonance. **a3** Superimposed stability (*blue curves*) and resonance (*red curves*) diagrams showing that intrinsic oscillations and resonance may occur in the absence of the other. **b** Natural ($f_{\mathrm{nat}}$), resonant ($f_{\mathrm{res}}$), and phase-resonant ($f_{\mathrm{phas}}$) frequencies as a function of *α* (**b1**) and *ϵ* (**b2**) illustrating that these characteristic frequencies have different values (**b1** and **b2**) and different monotonic properties (**b2**) as the model parameters change. **c** Maximum impedance ($Z_{\max}$) and resonance amplitude ($Q_{Z}$) as a function of *α* (**c1**) and *ϵ* (**c2**) illustrating the two basic mechanisms of generation of resonance in 2D linear systems. **c1** As *α* increases, resonance results from a decrease in both Z(0) and $Z_{\max}$ with Z(0) decreasing faster than $Z_{\max}$. **c2** As *ϵ* decreases (time-scale separation increases), resonance results from an increase in $Z_{\max}$ with Z(0) fixed

The stability diagram in the *α*–*ϵ* parameter space is presented in Fig. 3a1. From (19), the fixed point is a focus if ${\left(1-\epsilon \right)}^{2}-4\alpha \epsilon <0$ and a node otherwise. Foci are stable if 1+ϵ>0. Nodes are stable if 1+ϵ>0 and ϵ(1+α)>0. If ϵ(1+α)<0 the fixed point is a saddle. The natural frequency of the damped oscillations (for stable foci) is given by

(20) $$f_{\mathrm{nat}}=\frac{T}{2\pi }\mu \;\text{with }\mu =\frac{\sqrt{4\alpha \epsilon -{\left(1-\epsilon \right)}^{2}}}{2}.$$

#### 3.1.2 Impedance, Phase, Resonance, and Phase-Resonance

The impedance function (17) for system (14)–(15) with $A_{\mathrm{in}}>0$ is given by (Appendix A)

(21) $$Z^{2}(\Omega )=\frac{\epsilon^{2}+\Omega^{2}}{{\left[\epsilon (1+\alpha )-\Omega^{2}\right]}^{2}+{\left(1+\epsilon \right)}^{2}\Omega^{2}}.$$

The resonant frequency, if it exists, is given by

(22) $$f_{\mathrm{res}}=\frac{T}{2\pi }\Omega_{\mathrm{res}}\;\text{with }\Omega_{\mathrm{res}}=\sqrt{-\epsilon^{2}+\sqrt{\epsilon^{2}\alpha (\alpha +2\epsilon +2)}}.$$

System (14)–(15) exhibits resonance if $\Omega_{\mathrm{res}}>0$. In order $\Omega_{\mathrm{res}}$ to be defined, α(α+2ϵ+2)≥0 and $\sqrt{\epsilon^{2}\alpha (\alpha +2\epsilon +2)}>\epsilon^{2}$. The first condition is satisfied either if

(23) α≥−2(1+ϵ)(α≥0)orα<−2(1+ϵ)(α<0).

For the second condition to be satisfied,

(24) $$|\alpha +1+\epsilon |>\sqrt{2\epsilon^{2}+2\epsilon +1}.$$

This yields either

(25) $$\alpha >-1-\epsilon +\sqrt{2\epsilon^{2}+2\epsilon +1}\;\text{or}\;\alpha <-1-\epsilon -\sqrt{2\epsilon^{2}+2\epsilon +1}.$$

These regions are illustrated in the resonance diagram in Fig. 3a2. Figure 3a3 illustrates that resonance may occur in the absence of intrinsic oscillations and vice versa.

The phase *ϕ* for system (14)–(15) is given by

(26) $$\phi (\Omega )=\tan^{-1}\left(\frac{\Omega^{2}-\epsilon (\alpha -\epsilon )}{(\epsilon (\alpha +1)-\Omega^{2})\epsilon +(1+\epsilon )\Omega^{2}}\Omega \right).$$

The phase-resonant frequency, if it exists, is given by

(27) $$f_{\mathrm{phas}}=\frac{T}{2\pi }\Omega_{\mathrm{phas}}\;\text{with }\Omega_{\mathrm{phas}}=\sqrt{\epsilon (\alpha -\epsilon )}.$$

The natural, resonant, and phase-resonant frequencies do not necessarily coincide (Fig. 3b). In fact, they rarely do so.

Figure 3c illustrates the two basic mechanisms of generation of resonance for 2D linear systems [29] in terms of *α* and *ϵ*: (i) as *α* increases, resonance emerges from a combined and unbalanced decrease in both Z(0) and $Z_{\max}$ with Z(0) decreasing faster than $Z_{\max}$ (panel c1), and (ii) as *ϵ* decreases (time-scale separation between *v* and $w_{1}$ increases), resonance emerges from an increase in $Z_{\max}$ with Z(0) unchanged.

### 3.2 The Structure of the “Oscillatory Phase Plane”

Here we consider system (14)–(15) with $A_{\mathrm{in}}\geq 0$. For the type of analysis we present in this paper it is useful to rescale time by defining

(28) $$\hat{t}=ft$$

in order to separate the effect of the input frequency *f* from the input’s time dependence. Substituting (28) into (14)–(15), and dropping the “hat” sign from the time $\hat{t}$, we obtain

(29) $$\frac{dv}{dt}=\frac{1}{f}[-v-w+A_{t}],$$

(30) $$\frac{dw}{dt}=\frac{\epsilon }{f}[\alpha v-w],$$

where the sinusoidal input

(31) $$A_{t}=A_{\mathrm{in}}\sin(2\pi t/T)$$

has the same frequency (1 cycle per *T* units of time) for all values of the input frequency *f*. The latter affects the speed of the trajectories in the phase plane (i.e., the speed of the system’s response) without affecting the direction of the underlying vector field. Here we use T=1000.

The *v*- and *w*-nullclines for the unforced system ($A_{\mathrm{in}}=A_{t}=0$) are given by $N_{v}(v)=-v$ and $N_{w}(v)=\alpha v$, respectively. The stability properties of this autonomous system have been discussed in Sect. 3.1.1. For $A_{\mathrm{in}}>0$, system (29)–(30) is no longer two-dimensional. In our analysis, we will think of the projection of the zero-level curve for (29) for each value of *t* as the *v*-nullcline $N_{v}(v)$ for the autonomous system forced by the sinusoidal input $A_{t}$:

(32) $$N_{v,t}(v)=-v+A_{t}.$$

For t=0 (or any multiple of t=500), $N_{v,t}$ coincides $N_{v}=-v$. As *t* increases $N_{v,t}$ “moves” cyclically, generating lines parallel to $N_{v}=-v$ in between the lines

(33) $$N_{v}^{+}(v)=-v+A_{\mathrm{in}}\;\text{and}\;N_{v}^{-}(v)=-v-A_{\mathrm{in}}.$$

The moving *v*-nullcline reaches these two lines at t=250 and t=750, respectively. As $N_{v,t}$ moves so does its intersection with the *w*-nullcline $N_{w}$ generating a “moving fixed point”

(34) $$({\bar{v}}_{t},{\bar{w}}_{t})=\left(\frac{A_{t}}{1+\alpha },\frac{\alpha A_{t}}{1+\alpha }\right),$$

which oscillates with frequency 1 between the endpoints $\left({\bar{v}}_{250},{\bar{w}}_{250}\right)$ ($A_{250}=A_{\mathrm{in}}$) and $\left({\bar{v}}_{750},{\bar{w}}_{750}\right)$ ($A_{750}=-A_{\mathrm{in}}$), reaching the origin three times within a cycle at t=0, t=500, and t=1000.

This moving fixed point together with the moving nullclines organize the dynamics of the forced system. Specifically, the stable fixed point (either a node or a focus) acts as a moving target for trajectories, which track its motion by evolving with a *f*-dependent speed.

The dynamics of the forced system (29)–(30) results from the interaction between the vector field of the autonomous system ($A_{\mathrm{in}}=A_{t}=0$) and the forcing term ($A_{t}$) that causes the cyclic motion of both the *v*-nullcline and the fixed point (34) in addition to a cyclic change in the direction field. For each point in the phase plane the value of the direction field is independent of *f*. However, the trajectories’ *f*-dependent speed causes them to sweep different distances in a given unit of time. Since they reach different points, subject to different values (magnitude and direction) of the vector field, trajectories describe different curves for different values of *f*. The resonant frequency, if it exists, is the value of the input frequency *f* for which the corresponding limit cycle trajectory has the maximal amplitude in the *v*-direction, provided this amplitude is larger than the instantaneous amplitude corresponding to the effective resistance Z(0).

### 3.3 Transient Dynamics for Instantaneously Forced Systems

As a first step in our investigation we describe the response of system (29)–(30) to instantaneous constant inputs (with no associated time scales) in order to understand how the system’s intrinsic time scales depend on the parameters *α* and *ϵ*, and how these parameters contribute to the amplification or attenuation of the voltage response to these instantaneous changes. We will use this knowledge to explain the dynamics of the forced system when the inputs do have associated times scales.

For the purpose of this part we will consider constant values of $A_{t}$ in system (29)–(30). Without loss of generality, we assume that (i) $A_{t}$ instantaneously changes from $A_{t}=0$ to either $A_{t}=1$ or $A_{t}=-1$, (ii) this change occurs at t=0, and (iii) f=1.

#### 3.3.1 Transient Dynamics and Effective Time Scales

An instantaneous change in $A_{t}$ causes an instantaneous displacement of the *v*-nullcline on the phase plane, and consequently an instantaneous change in the location of the fixed point. The vector field changes accordingly, and can also be thought of being instantaneously displaced.

The stability properties of the fixed point, however, remain unchanged since the system (29)–(30) is linear and, therefore, its associated Jacobian matrix is independent of $A_{t}$.

**Fig. 4:**
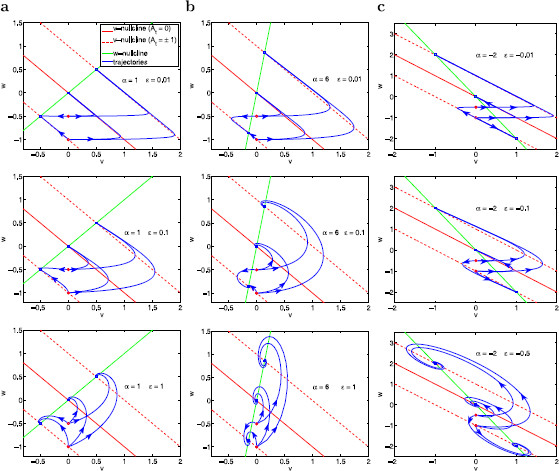
Phase-plane diagrams for the autonomous linear system (29)–(30) for various representative values of *α*, *ϵ* and $A_{t}$. **a**α=1. *Top row*: ϵ=0.01. *Middle row*: ϵ=0.1. *Bottom row*: ϵ=1. **b**α=6. *Top row*: ϵ=0.01. *Middle row*: ϵ=0.1. *Bottom row*: ϵ=1. **c**α=−2. *Top row*: ϵ=−0.01. *Middle row*: ϵ=−0.1. *Bottom row*: ϵ=−0.5. Each *panel* shows superimposed phase-planes diagrams for three different constant values of $A_{t}$ (=0,1,−1) generating three *v*-nullclines (*solid-red* for $A_{t}=0$, *dashed-red* for $A_{t}=1$ and $A_{t}=-1$) and three fixed points (*blue dots* on the intersections between the *red* and *green lines*). The *w*-nullcline (*green line*) is common to all values of $A_{t}$. *Solid-red line*: *v*-nullclines for $A_{t}=0$. *Dashed-red lines*: *v*-nullclines for $A_{t}=1$ (*above*) and $A_{t}=-1$ (*below*). *Red dots* at (0,−1) and (0,−0.5): representative initial conditions. *Solid-blue lines*: trajectories initially located at these initial points. Each trajectory emerging from *the red dots* corresponds to a different value of $A_{t}$ and converges to the corresponding fixed point. The fixed points in panels **a**-*top*, **a**-*middle* and **b**-*top* are stable nodes and the fixed points in panels **a**-*bottom*, **b**-*middle* and **b**-*bottom* are stable foci

Consequently, for different values of $A_{t}$, trajectories at a given initial point have the same long term behavior as they approach the corresponding fixed points, but they have different initial transient behaviors (Fig. 4). For the purpose of this paper, we are particularly interested in the initial transient behavior caused by changes in $A_{t}$. As we explain in more detail below, this transient behavior rather than the long term behavior is the one that plays an important role in determining the amplitudes of the limit cycle trajectories for the time-dependent forced systems. 

The trajectories’ transient behavior relevant to this paper corresponds to the time elapsed from the initial point (red dots in Fig. 4) until they cross the corresponding *v*-nullcline (dashed-red line), reaching their maximum value for *v*. The effect of different values of $A_{t}$ on the behavior of trajectories initially at the same point can be understood by looking at the horizontal and vertical components of the vector field

(35) $$D_{v}=-v-w+A_{t}\;\text{and}\;D_{w}=\epsilon (\alpha v-w),$$

respectively. As $A_{t}$ increases, $D_{v}$ increases and the motion in the horizontal direction strengthens relative to the motion in the vertical direction. Consequently, the larger $A_{t}$ the “more horizontal” the trajectories’ transient direction of motion. From a different perspective, trajectories starting at the same initial point in the “standard” coordinate system whose origin is (0,0), start at different initial points in translated coordinate systems whose origins are the fixed points $\left({\bar{v}}_{t},{\bar{w}}_{t}\right)$ determined by $A_{t}$ through (34). The larger $A_{t}$, the larger the distance between the trajectory’s initial point and the corresponding *v*-nullcline, and so they are less affected by it and they can transiently move in more horizontal directions.

Figure 4 also illustrates the roles of *α* and *ϵ* in determining both the effective time scales for system (29)–(30) and the amplitude of the voltage response (maximum value of *v* for any given trajectory) to constant perturbations ($A_{t}$). For ϵ=0.01 (top panels) the time scales are well separated and the system is fast-slow. Trajectories move first fast in almost horizontal directions, then they slow down as they approach the *v*-nullcline, and finally they reverse direction and move towards the fixed point in close vicinities of the displaced *v*-nullcline. This effect is more pronounced for smaller values of *α* (compare top panels a and b) because of the product *αϵ* in (30), which affects the effective time scales. As *α* decreases so does the slope of the *v*-nullcline, thus “pressing” trajectories that become more “compacted”. As *ϵ* increases (from the top to the bottom panels), the separation of time scales decreases, trajectories become more “rounded”, and reach lower maximal values of *v*.

**Fig. 5:**
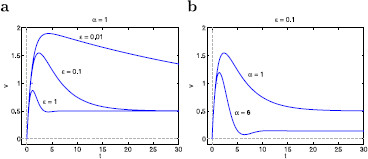
Voltage traces for the autonomous linear system (29)–(30) for various representative values of *α*, *ϵ* and $A_{t}=1$. **a**α=1. The maximum times are $t_{\max,\epsilon =0.01}=4.31$, $t_{\max,\epsilon =0.1}=2.38$, and $t_{\max,\epsilon =1}=1.12$. **b**ϵ=0.1. The maximum times are $t_{\max,\alpha =1}=2.38$ and $t_{\max,\alpha =6}=1.44$

An important observation is that, qualitatively, the initial transient behavior of trajectories in Fig. 4 is not dependent on whether the fixed point is a stable node or focus but rather on the magnitude of the differences between the corresponding values of *α* and *ϵ*. Specifically, trajectories with close enough values of both *α* and *ϵ* will display qualitatively similar transient behavior regardless of the possible differences in the stability of the corresponding fixed points (Fig. 5). 

#### 3.3.2 Amplification of the Voltage Response to Instantaneous Constant Inputs

The voltage response is amplified by a given parameter if *v* is able to reach a higher value when this parameter changes. Figures 4 and 5 demonstrate that the amplification of the voltage response increases as the time-scale separation increases (*ϵ* decreases) (compare top, middle and bottom panels for increasing values of *ϵ* in Fig. 4 and see the traces in Fig. 5) and as *α* decreases (compare Figs. 4a and 4b and see the traces in Fig. 5). These results reflect the roles of the resonant and amplifying currents in determining the amplification of the voltage response through the dimensionless parameters *α* and *ϵ* given by (12). For example, an increase in time constant $\tau_{1}$ causes a decrease in *ϵ* (time-scale separation), leading to an amplification of the voltage response. The explanation of the effects of other biophysical parameters (e.g., $g_{L}$ and $g_{1}$) through the dimensionless parameter *α* is less straightforward since *α* has been used as a voltage scale. A thorough study of these effects has been carried in [29]. 

Figure 4 also demonstrates that voltage responses are more amplified for negative values of *α* and *ϵ* than for positive ones (compare panels a and c). This is consistent with the fact that negative values of *α* and *ϵ* are obtained for negative values of the effective conductance $g_{L}$, which in turn reflects the presence of a strong, fast amplifying current ($I_{2}$) in the biophysical model.

### 3.4 Voltage Amplitude Response to Sinusoidal Inputs at Different Frequencies: Resonance and Phase-Resonance

Here we build up on the ideas discussed in Sect. 3.3 to investigate the mechanism of selection of the resonant and phase-resonant frequencies in the two-dimensional system (29)–(30) in response to sinusoidal inputs of the form $A_{t}$ (31). Without loss of generality we consider the canonical case $A_{\mathrm{in}}=1$. With this choice, for each value of *f* the impedance function (18) coincides with the maximum value of *v*; i.e., $v_{\max}(f)=Z(f)$.

As *t* increases, $A_{t}$ changes and the *v*-nullcline $N_{v,t}$ (32) moves cyclically, parallel to itself between the lines $N_{v}^{+}(v)$ and $N_{v}^{-}(v)$ (33) (dashed-red lines in Fig. 4) with frequency equal to 1. The fixed point (34) moves cyclically with the same frequency between the two corresponding extreme values. The input amplitude is coded by the distance between the moving fixed point and the origin.

Trajectories respond to changes in $A_{t}$ by evolving according to the dynamics dictated by the underlying vector field (35). The trajectories’ speed *ν* is given by the product of the magnitude of the vector field and $f^{-1}$

(36) $$\nu =\frac{\sqrt{D_{v}^{2}+D_{w}^{2}}}{f}=\frac{\sqrt{{\left(-v-w+A_{t}\right)}^{2}+\epsilon^{2}{\left(\alpha v-w\right)}^{2}}}{f}.$$

**Fig. 6:**
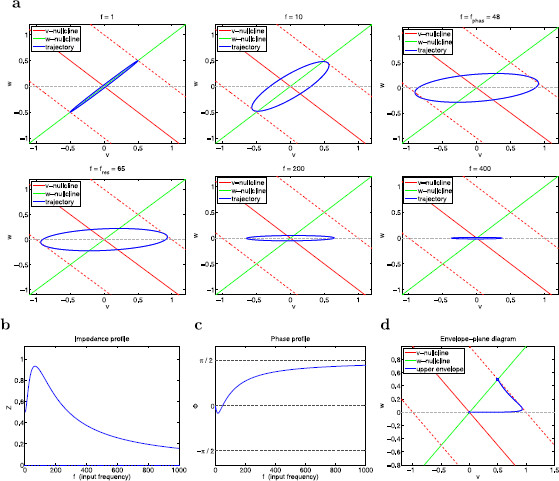
Dynamics of the sinusoidally forced linear system (29)–(30) for α=1, ϵ=0.01. **a** Projections of the phase-plane diagrams on the *v*–*w* plane for various representative values of the input frequency *f*. *The fixed solid-red line* and *the solid-green line* represent the *v*- and *w*-nullclines for the unforced system ($A_{t}=0$), respectively. *The dashed-red lines* represent the *v*-nullclines for $A_{t}=1$ (*above-right*) and $A_{t}=-1$ (*below-left*). *The solid-blue lines* represent the trajectories of the forced system for a single period (T=1000). **b** Impedance profile. **c** Phase profile. **d** Envelope-plane diagram. *The solid-blue line* represents the envelope curve: Each point on this curve is the maximum point on the limit cycle response to sinusoidal inputs parametrized by the input frequency *f* which increases from f=0 (*blue-square* at the intersection between upper *dashed-red* and *green curves*) to f→∞ (*blue dot* at the origin). (The *v*-coordinates of the envelope curve are the impedance function *Z*, since $A_{\mathrm{in}}=1$.) *Solid-red* and *-green lines*: *v*- and *w*-nullclines for the unforced system ($A_{t}=0$). *Dashed-red lines*: *v*-nullclines for $A_{t}=1$ (*above*) and $A_{t}=-1$ (*below*)

At any given point, the underlying vector field is independent of *f*. However, the *f*-dependent speed (36) causes the resulting limit cycle trajectory to be *f*-dependent as explained at the end of Sect. 3.2. This dependence is reflected primarily on the shape and orientation of the limit cycle as we illustrate this in Fig. 6a for a system that exhibits resonance (see Fig. 6b) and phase-resonance (see Fig. 6c). For small values of *f* limit cycles are elliptic and very narrow, with their major axis lying on a vicinity *w*-nullcline. As *f* increases, the limit cycle first widens and rotates, then it narrows as it continues to rotate until the major axis becomes horizontal (on the *v*-axis), and finally the limit cycle shrinks until it collapses to a point as f→∞. The system exhibits resonance because the maximal value of *v* for f∼65 is larger than the maximal value of *v* for f→0. 

#### 3.4.1 Envelope-Plane Diagrams: Resonant and Phase-Resonant Responses

Figure 6d shows a graph containing the curve generated by the points with maximum values of *v* on the limit cycles for continuously changing values of f∈[0,∞) and the parameter values in Fig. 6a. We refer to this curve as the upper envelope curve of the voltage response. (The lower envelope curve, not shown, is determined by the minimum values of *v* on the *f*-dependent limit cycles, and is symmetric to the upper envelope curve with respect to the *v*-nullcline and *w*-nullclines.) We refer to the diagrams containing the envelope curves together with the *v*- and *w*-nullclines (green, solid-red, and dashed-red), as in Fig. 6d, as the *envelope-plane diagrams*.

Envelope-plane diagrams contain geometric and dynamic information about a system’s frequency response to oscillatory inputs, and they are the frequency analogs to phase-plane diagrams. Trajectories in the envelope-plane diagrams (upper and lower envelopes) are curves parameterized by the input frequency as trajectories in the phase planes are curves parametrized by time. Neither *f* nor *t* are explicit in the corresponding diagrams. The red-dashed lines quantify the maximal input amplitude. For f→0, the envelope curve is at the intersection between the upper dashed-red line $N_{v}^{+}$ and the *w*-nullcline. For f→∞, the envelope curve is at the origin (limit cycle shrinking to a point).

The cusp (“horizontal peak”) in the envelope curve in Fig. 6d corresponds to the peak in the impedance profile (Fig. 6b), and hence to the resonant frequency. At this cusp the voltage response is the largest across input frequencies, and larger than the response for f→0: $v_{\max}={\left(1+\alpha \right)}^{-1}$.

The point in the envelope curve tangent to the upper dashed-red line corresponds to the phase-resonant frequency for reasons that we will clarify later in the paper.

Below, we first discuss the voltage response mechanisms for the two limiting cases (f→0 and f→∞) and then elaborate on the dynamics for intermediate values of *f*, in particular $f_{\mathrm{res}}$ and $f_{\mathrm{phas}}$.

#### 3.4.2 Low Input Frequencies Generate Quasi-one-dimensional Dynamics Along the *w*-Nullcline

**Fig. 7:**
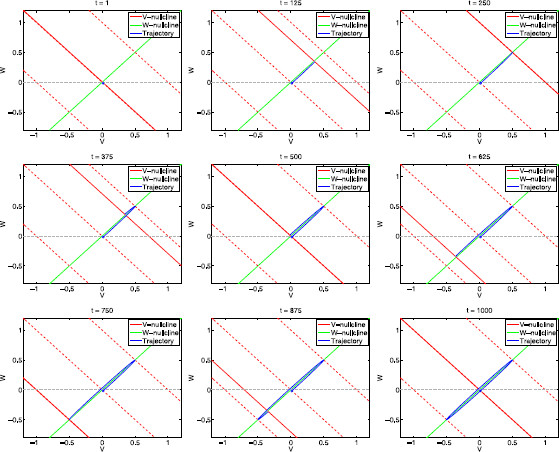
Snapshots of the moving phase-plane diagram for the linear system (29)–(30) for α=1, ϵ=0.1, f=1 and representative values of *t* within one cycle (T=1000). *The solid-red lines* represent the moving *v*-nullcline. *The dashed-red lines* represent the *v*-nullclines for $A_{t}=0$ (*middle*), $A_{t}=1$ (*top*), and $A_{t}=-1$ (*bottom*). *The solid-green line* represents the (fixed) *w*-nullcline. *The fixed solid-red line* and *the solid-green line* represent the *v*- and *w*-nullclines for the unforced system ($A_{t}=0$), respectively. *The dashed-red lines* represent the *v*-nullclines for $A_{t}=1$ (*above*), $A_{t}=0$ (*middle*), and $A_{t}=-1$ (*below*). As *t* increases, the red nullcline moves cyclically between *the two dashed-red lines*. *The blue dot* indicates the initial location of the trajectory (t=0=1000)

For values of f≪1 both equations in system (29)–(30) are very fast (dv/dt→∞ and dw/dt→∞), and then the limit cycle trajectory tracks the motion of the fixed point almost instantaneously (in a quasi-steady-state fashion). In the limit f→0, the limit cycle trajectory moves cyclically along the *w*-nullcline in between the points (34) with $A_{t}=\pm A_{\mathrm{in}}=\pm 1$. From (34), $v_{\max}/A_{\mathrm{in}}={\left(1+\alpha \right)}^{-1}$, which coincides with Z(0) in (21). In Fig. 7 we present snapshots of the evolution of the limit cycle trajectory for f=1 (corresponding to Fig. 6a, top-left panel). 

#### 3.4.3 High Input Frequencies Generate Quasi-one-dimensional Dynamics Along the Horizontal *v*-Axis

For large enough values of f≫1, both (29) and (30) are very slow (dv/dt→0 and dw/dt→0), and then the limit cycle trajectory evolves with a very low speed according to (36). Relative to the trajectory’s speed, the *v*-nullcline $N_{v,t}$ moves very fast. The time it takes the trajectory to cover a very small distance while tracking the motion of the *v*-nullcline is enough for the latter to reach its maximum level $N_{v}^{+}$, reverse direction, and intersect the trajectory on its “way back”. This causes the trajectory to reverse direction. As a result, the limit cycle trajectory is not able to cover any significant distance away from the origin, and so the amplitude of the limit cycle is small as compared to other values of *f* (Fig. 6a, bottom-right panel). In the limit f→∞, the amplitude of the limit cycle trajectory is zero (the limit cycle shrinks to the origin), which coincides with $\lim_{f\to \infty }Z(f)=0$.

#### 3.4.4 Voltage Response Amplification at the Resonant Frequency Band

For small and large enough values of *f* the corresponding limit cycle trajectories are constrained to move along quasi-one-dimensional directions (*w*-nullcline and *v*-axis, respectively). For intermediate values of *f* there is a transition in the shapes of the limit cycle trajectories between these two limit cases. Specifically, limit cycle trajectories are neither too fast nor too slow, and then, while they are “left behind” by the moving *v*-nullcline, they can take advantage of the two-dimensional vector field without being constrained to move in quasi-one-dimensional directions. For the appropriate values of *α* and *ϵ* this degree of freedom allows limit cycle trajectories to reach values of $v_{\max}(f)$ larger than $v_{\max}(0)$, and so to exhibit resonance.

**Fig. 8:**
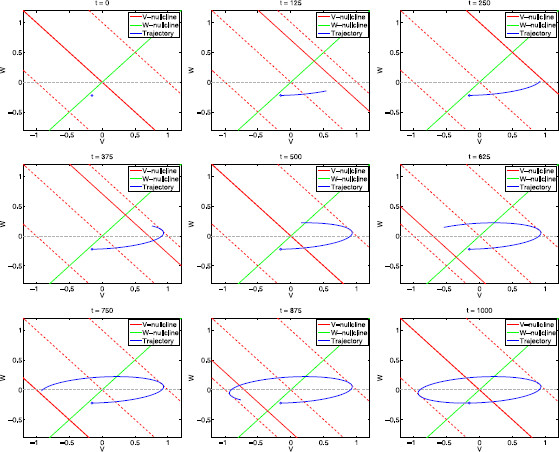
Snapshots of the moving phase-plane diagram for the linear system (29)–(30) for α=1, ϵ=0.1, f=65 and representative values of *t* within one cycle (T=1000). *The solid-red lines* represent the moving *v*-nullcline. *The dashed-red lines* represent the *v*-nullclines for $A_{t}=0$ (*middle*), $A_{t}=1$ (*top*), and $A_{t}=-1$ (*bottom*). *The solid-green line* represents the (fixed) *w*-nullcline. *The fixed solid-red line* and *the solid-green line* represent the *v*- and *w*-nullclines for the unforced system ($A_{t}=0$), respectively. *The dashed-red lines* represent the *v*-nullclines for $A_{t}=1$ (*above*), $A_{t}=0$ (*middle*), and $A_{t}=-1$ (*below*). As *t* increases, the red nullcline moves cyclically between *the two dashed-red lines*. *The blue dot* indicates the initial location of the trajectory (t=0=1000)

In Fig. 8 we show a sequence of snapshots of the evolution of the limit cycle trajectory for $f=f_{\mathrm{res}}=65$ (Fig. 6a, bottom-left). The initial snapshot (t=0) corresponds to a point (blue dot) on the limit cycle trajectory and $A_{t}=0$. As *t* increases the *v*-nullcline moves and the limit cycle trajectory tracks its motion. The limit cycle’ shape and amplitude result from the combined effect of $A_{t}$, *f*, and the model parameters (*α* and *ϵ*). 

In Fig. 8 we are using a relatively small value of *ϵ* (ϵ=0.1). While, small values of *ϵ* are enough to account for the almost horizontal transient trajectories in autonomous systems (Fig. 4), they are not enough to account for the almost horizontal directions of motion for limit cycles trajectories in forced systems such as in Fig. 8. In fact, for the same value of *ϵ* but a different value of *f* (f=1 instead of f=65) the limit cycle trajectory moves in the (oblique) direction of the *w*-nullcline (Fig. 7).

To better understand how $A_{t}$, *f*, *ϵ* and *α* affect the direction of motion of the limit cycle trajectory in Fig. 8, it is useful to go back and look at the initial transient segment of the evolution of trajectories for the autonomous systems presented in Fig. 4 (from the initial, red point until they cross the *v*-nullcline). Figure 4a (middle) corresponds to the same parameter values (α=1 and ϵ=0.1) as in Fig. 8. The increase in the constant input from $A_{t}=0$ to $A_{t}=1$ causes both an increase in the distance between the tip of the trajectory and the *v*-nullcline (from the solid line to the dashed-red line) and a strengthening of the horizontal component of the vector field $D_{v}$ (35) relative to its vertical component $D_{w}$. As a result, during the initial transient interval, the larger $A_{t}>0$, the more horizontal is the trajectory’s direction of motion.

A similar, but dynamic effect is partially responsible for the determination of the direction of motion in Fig. 8. Trajectories move faster in more horizontal directions as $A_{t}$ increases during the ascending phase (t=0 to t=250) because the continuous increase in $A_{t}$ causes a continuous increase in $D_{v}$ while $D_{w}$ remains almost unchanged. However, differently from the static case, in the dynamic case there is an opposite, dynamic effect caused by the decreasing distance between the tip of the limit cycle trajectory and the moving *v*-nullcline $N_{v,t}$ since $D_{v}$ decreases as the limit cycle trajectory approaches the *v*-nullcline.

In the autonomous case, the nullclines are fixed, so the distance between the trajectory and $N_{v,t}$ depends only on the trajectory’s speed (36). In the forced case, on the other hand, the trajectory and the *v*-nullcline $N_{v,t}$ are simultaneously moving, with different speeds. The resonance frequency $f_{\mathrm{res}}$ is the input frequency for which their relative speed is optimal in the sense that it allows the limit cycle trajectory to move in a direction that maximizes (over the range of input frequencies *f*) the distance (in the *v* direction) the limit cycle trajectory can cover before intersecting the moving *v*-nullcline, at which time they are forced to reverse direction. This causes a maximization (over the range of input frequencies *f*) of the maximum value $v_{\max}$ on the limit cycle trajectories. In Fig. 8 ($f∼f_{\mathrm{res}}$), this intersection occurs for $v_{\max}$ (∼1). The remainder of the limit cycle can be explained using similar ideas.

For values of $f<f_{\mathrm{res}}$, the limit cycle trajectory moves faster than for $f=f_{\mathrm{res}}$, and so the direction of motion is less horizontal causing this cycle trajectory to intersect $N_{v,t}$ at a higher point; i.e., for a lower value of $v_{\max}$ (e.g., Fig. 6a, f=48). For values of $f>f_{\mathrm{res}}$, the limit cycle trajectory moves slower than for $f=f_{\mathrm{res}}$. The relative speed between this trajectory and $N_{v,t}$ is large enough to allow the limit trajectory to move in an almost horizontal direction, but, due to the limit cycle trajectory’s lower speed, $N_{v,t}$ “returns” before this trajectory covers a large enough distance. Thus, the intersection between the trajectory and $N_{v,t}$ occurs for lower values of $v_{\max}$ (e.g., Fig. 6a, f=200).

#### 3.4.5 Resonance and Intrinsic Oscillations Are Generated by Related, but not Identical Mechanisms

Intrinsic STOs and subthreshold resonance have been proposed to result from the same underlying mechanism [12]. However, in linear systems, resonance may occur in the absence of intrinsic oscillations and vice versa [11] (see Fig. 3a). Even for parameter values for which linear systems exhibit both resonance and intrinsic oscillations, the resonant and natural frequencies do not necessarily coincide (Fig. 3b).

The phase-plane analysis discussed above demonstrates that the resonant properties are not expected to be directly linked to the stability properties of the underlying autonomous system. The time scales corresponding to intrinsic oscillations, if they exist, are determined by the stability properties of the stable foci, which reflect the long term behavior of trajectories. In contrast, the time scales corresponding to the resonant frequency do not involve the stability properties of the fixed points of the underlying autonomous system (nodes or foci) but rather the initial transient behavior of trajectories as described above. This initial transient behavior is the link between the autonomous and forced systems. This transient dynamics is associated with either the onset of intrinsic oscillations (foci) or a “sag” (node) typically observed in the response of h-currents to constant inputs [11]. Importantly, as we demonstrated in Sect. 3.3, autonomous systems may display qualitatively similar transient behavior for nodes and foci, even though the long time behavior of the corresponding trajectories is qualitatively different (see Fig. 4b).

#### 3.4.6 Phase Advance, Phase Delay and Phase-Resonance

In Fig. 3b we showed that the resonant ($f_{\mathrm{res}}$) and phase-resonant ($f_{\mathrm{phas}}$) frequencies do not necessarily coincide, and that resonance may occur in the absence of phase-resonance. For the parameters values in Fig. 6, $f_{\mathrm{res}}=65$ and $f_{\mathrm{phas}}=48$. Typical phase profiles show “phase advance” for $f<f_{\mathrm{phas}}$ and “phase delay” for $f>f_{\mathrm{phas}}$ (see Fig. 6c).

Geometrically, for the output and input to be synchronized in phase, the intersection between the limit cycle trajectory and the moving *v*-nullcline must occur when the *v*-nullcline reaches its highest level $A_{t}=A_{\mathrm{in}}$ (when the solid-red line in the moving phase-plane diagrams reaches the dashed-red line at t=250). The phase is advanced when the limit cycle trajectory “peaks” before the input does, which requires the trajectory to evolve fast enough as compared to the speed of the *v*-nullcline. Conversely, the phase is delayed when the limit cycle trajectory intersects the *v*-nullcline “on its way back” (descending phase), after the *v*-nullcline has reached its highest level. For $f=f_{\mathrm{res}}=65$ (Fig. 8), the limit cycle trajectory is still behind the *v*-nullcline at the time this *v*-nullcline reaches its highest level (t=250), and the phase is therefore delayed (the intersection occurs at a time in between the top-right and middle-left panels, t=250 and t=375, respectively).

**Fig. 9:**
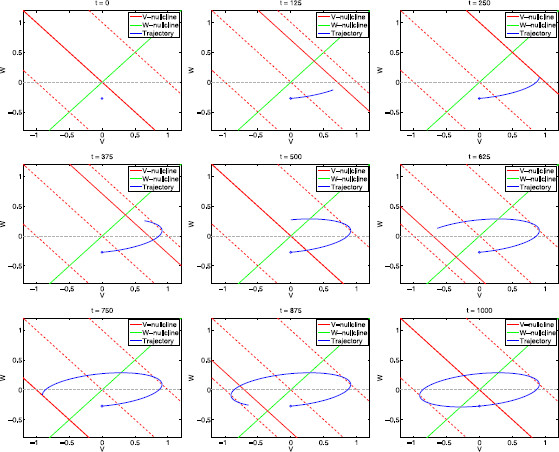
Snapshots of the moving phase-plane diagram for the linear system (29)–(30) for α=1, ϵ=0.1, f=48 and representative values of *t* within one cycle (T=1000). *The solid-red lines* represent the moving *v*-nullcline. *The dashed-red lines* represent the *v*-nullclines for $A_{t}=0$ (*middle*), $A_{t}=1$ (*top*), and $A_{t}=-1$ (*bottom*). *The solid-green line* represents the (fixed) *w*-nullcline. *The fixed solid-red line* and *the solid-green line* represent the *v*- and *w*-nullclines for the unforced system ($A_{t}=0$), respectively. *The dashed-red lines* represent the *v*-nullclines for $A_{t}=1$ (*above*), $A_{t}=0$ (*middle*), and $A_{t}=-1$ (*below*). As *t* increases, the red nullcline moves cyclically between *the two dashed-red lines*. *The blue dot* indicates the initial location of the trajectory (t=0=1000)

For $f=f_{\mathrm{phas}}=48$ (Fig. 9), on the other hand, the limit cycle trajectory intersects the *v*-nullcline when the latter is at its highest level (t=250). Since the point of intersection corresponds to $v=v_{\max}$ (on the limit cycle trajectory), the corresponding point on the envelope curve “touches” the top dash-red line. For all other frequencies, the intersection between limit cycle trajectories and the moving *v*-nullcline occurs away from the top dashed-red line, therefore the phase-resonant frequency corresponds to the point in the envelope curve that is tangent to the top dashed-red line. This tangent point is not necessarily the cusp that corresponds to the resonance frequency $f_{\mathrm{res}}$. 

Specifically, the phase-resonance phenomenon depends on the ability of the limit cycle trajectory for $f=f_{\mathrm{phas}}$ to track the *v*-nullcline fast enough so to reach $v_{\max}$ before the *v*-nullcline reaches its highest level. But this does not preclude limit cycle trajectories for frequencies $f\neq f_{\mathrm{phas}}$ to reach higher values of *v* due to the different directions of motion generated by these values of *f*. In fact, values of $f>f_{\mathrm{phas}}$ cause limit cycle trajectories to evolve slower than for $f=f_{\mathrm{phas}}$, and so (within some range of values of *f*) they can move along more horizontal directions, and thus reach higher values of *v* before intersecting the “returning” *v*-nullcline. This is the case for $f=f_{\mathrm{res}}$ in Fig. 8.

### 3.5 Effects of *α* and *ϵ* on Resonance, Phase-Resonance, and Other Attributes of the Impedance and Phase Profiles

Geometrically, the value of *α* determines one of the boundaries of the region (triangle) in the envelope-plane diagram where the envelope curves live (Fig. 6d). The other two boundaries are the *v*-nullcline for $A_{t}=1$ (dashed-red line) and the *v*-axis. The value of *α* also determines $Z(0)=v_{\max}(0)/A_{\mathrm{in}}={\left(1+\alpha \right)}^{-1}$, which is the *v*-coordinate of the intersection between the *w*-nullcline and the *v*-nullcline for $A_{t}=1$. As we mentioned earlier, for resonance to occur, $v_{\max}(f)>v_{\max}(0)$ (on the envelope curve) for some range of values of *f* around $f_{\mathrm{res}}$.

The value of *ϵ* plays a key role in determining the direction of motion of limit cycle trajectories analogous to its effect on the initial transient behavior of trajectories for the autonomous systems discussed in the context of Fig. 4. For fixed values of *α*, the smaller *ϵ* the more horizontal (and less rounded) is the direction of motion of the initial portion of the trajectories, and the larger the value of *v* these trajectories reach; i.e., the voltage response is amplified.

From the biophysical point of view, *α* and *ϵ* convey information about the effective conductances $g_{1}$ and $g_{L}$, the capacitance *C*, and the time constant ${\bar{\tau }}_{1}$ (gating variable $x_{1}$) through (12). In [29] we use numerical simulations to conduct a through analysis of the effects of resonant and amplifying biophysical conductances on the determination of $f_{\mathrm{res}}$, $f_{\mathrm{phas}}$ and other attributes of the impedance and phase profiles.

**Fig. 10:**
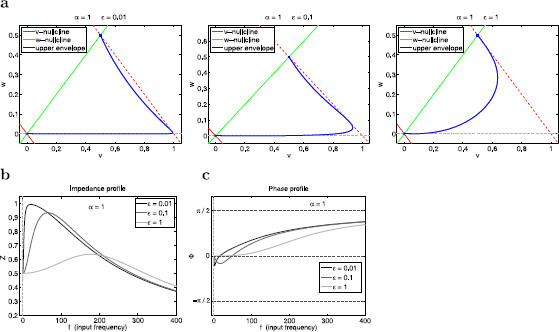
Effects of changes in *ϵ* on the resonant and phase-resonant properties of the linear system (29)–(30) for α=1. **a** Envelope-plane diagrams for ϵ=0.01 (*left*), ϵ=0.1 (*middle*), and ϵ=1 (*right*). **b** Impedance (*Z*) profiles. **c** Phase (*ϕ*) profiles. The parameter values correspond to the autonomous systems presented in Fig. 4a

**Fig. 11:**
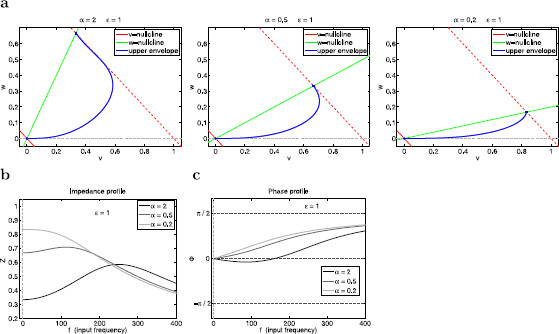
Effects of changes in *α* on the resonant properties of the linear system (29)–(30) for ϵ=1. **a** Envelope-plane diagrams for α=2 (*left*), α=0.5 (*middle*), and α=0.2 (*right*). **b** Impedance (*Z*) profiles. **c** Phase (*ϕ*) profiles

**Fig. 12:**
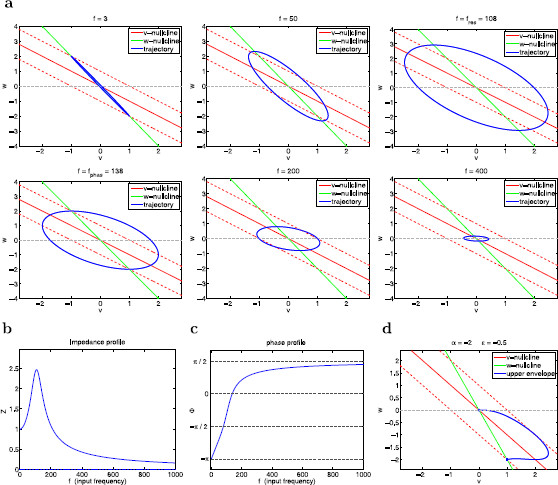
Dynamics of the sinusoidally forced linear system (29)–(30) for α=−2, ϵ=−0.5. **a** Projections of the phase-plane diagrams on the *v*–*w* plane for various representative values of the input frequency *f*. *The fixed solid-red line* and *the solid-green line* represent the *v*- and *w*-nullclines for the unforced system ($A_{t}=0$), respectively. *The dashed-red lines* represent the *v*-nullclines for $A_{t}=1$ (*above-right*) and $A_{t}=-1$ (*below-left*). *The solid-blue lines* represent the trajectories of the forced system for a single period (T=1000). **b** Impedance profile. **c** Phase profile. **d** Envelope-plane diagram. *The solid-blue line* represents the envelope curve: Each point on this curve is the maximum point on the limit cycle response to sinusoidal inputs parametrized by the input frequency *f* which increases from f=0 (*blue-square* at the intersection between *upper dashed-red* and *green curves*) to f→∞ (*blue dot* at the origin). (The *v*-coordinates of the envelope curve are the impedance function *Z*, since $A_{\mathrm{in}}=1$.) *Solid-red* and *-green lines*: *v*- and *w*-nullclines for the unforced system ($A_{t}=0$). *Dashed-red lines*: *v*-nullclines for $A_{t}=1$ (*above*) and $A_{t}=-1$ (*below*)

**Fig. 13:**
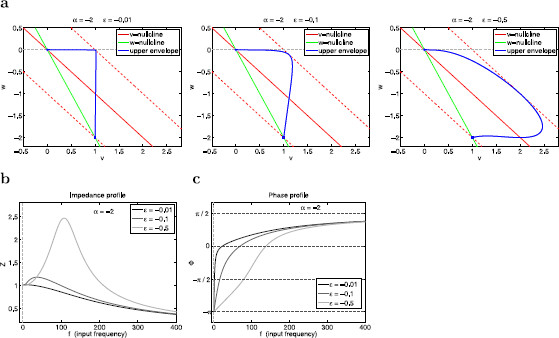
Effects of changes in *ϵ* on the resonant properties of the linear system (29)–(30) for α=−2. **a** Envelope-plane diagrams for ϵ=−0.01 (*left*), ϵ=−0.1 (*middle*), and ϵ=−0.5 (*right*). **b** Impedance (*Z*) profiles. **c** Phase (*ϕ*) profiles

Here we use the envelope-plain diagrams developed in Sect. 3.4 to explain how the parameters *α* and *ϵ* affect the resonant and phase-resonant properties of (14)–(15), and how they contribute to the amplification of the voltage response. In Figs. 10 and 11 we examine the effects of changes in *ϵ* and *α*, respectively. In Figs. 12 and 13 we investigate the additional amplifying effects for negative values of both *α* and *ϵ*. 

#### 3.5.1 An Increase in the Time-Scale Separation (Decreasing Values of *ϵ*) Causes an Amplification of the Voltage Amplitude Response

In Fig. 10 we compare the voltage responses for various representative values of *ϵ* and α=1. As *ϵ* decreases, both $Z_{\max}$ and $Q_{Z}$ increase, $f_{\mathrm{res}}$ and $f_{\mathrm{phas}}$ decrease, and the neuron becomes more selective ($\Lambda_{1/2}$ decreases) (Fig. 10b). These differences are captured by the shapes of the envelope curves, which are sharper the smaller the *ϵ*. For ϵ=0.01 (panel a, left) the system is fast-slow causing trajectories to move fast along horizontal fibers as compared to larger values of *ϵ* (middle and right panels), and thus the limit cycle trajectory corresponding to $f_{\mathrm{res}}$ reaches a higher value of $v_{\max}$ (the voltage response is more amplified). The envelope-plane diagrams predict that $f_{\mathrm{res}}$ and $f_{\mathrm{phas}}$ are very close since the envelope curve is tangent to the dashed-red line almost at the peak (panel a).

As *ϵ* increases, the tangent point and the peak separate (panels b and c), and hence the difference between $f_{\mathrm{res}}$ and $f_{\mathrm{phas}}$ increases. For ϵ=1 (panel c), the tangent point coincides with the intersection between the green and dashed-red lines, and hence the system exhibits no phase-resonance ($f_{\mathrm{phas}}=0$), while it still exhibits resonance.

In the limit of ϵ→0, the system becomes quasi-one-dimensional and the dynamics occurs almost exclusively along the *v*-axis for almost all values of *f*, except a small range close to f=0. For ϵ=0 the dynamics is fully one-dimensional, thus generating a low-pass filter response (no resonance). For f→0 (and ϵ=0) in (29) and (30) the maximum value of *v* approaches the intersection between the dotted-red line and the *v*-axis in the envelope-plane diagram. The maximum value of *v* (along the *v*-axis) decreases as *f* increases. As soon as ϵ>0, the dynamics becomes two-dimensional. The envelope curves unfold in the two-dimensional space and acquire “triangular-like” shapes similar to these shown in Fig. 10a (left), but peakier the smaller *ϵ*. Regardless of how small is *ϵ*, there is always a range of values of f<ϵ such that as f→0, the limit cycle trajectory evolves fast, “almost along” the *w*-nullcline (green line) following the motion of the fixed point. Therefore, the point on the envelope curve for f=0 lies on the intersection of the dotted-red line and the *w*-nullcline. The remaining of the envelope curve will be as in Fig. 10a, but the distance between the envelope curve and the dotted-red line will be larger the smaller *ϵ*. This distance is determined by the minimum phase, which increases in absolute value as *ϵ* decreases.

#### 3.5.2 Increasing Values of *α* Generate Resonance and Phase-Resonance and Cause an Amplification of the Voltage Response

In Fig. 11 we compare the voltage responses for various representative values of *α* and ϵ=1. The value of *α* determines the slope of the *w*-nullcline, which is steeper for larger values of *α*, and $v_{\max}(0)={\left(1+\alpha \right)}^{-1}$. The smaller slope of the *w*-nullcline for lower values of *α* (right panel) reduces the trajectories’ freedom of motion by constraining them to evolve in close vicinities of both nullclines (the *w*-nullcline and the moving *v*-nullcline). The consequent decrease in speed prevents the limit cycle trajectory from reaching large enough values of $v_{\max}$. This together with the fact that $v_{\max}(0)$ increases with *α* prevents the system from exhibiting resonance.

For larger values of *α* (left and middle panels), $v_{\max}(0)$ is smaller. Although an increase in *α* increases the horizontal component of the vector field (through the product *αϵ*) and hence reduces the effective time-scale separation, this is not enough to prevent limit cycle trajectories from moving beyond $v_{\max}(0)$, and the system is able to exhibit resonance.

The envelope-plane diagrams predict the generation of phase-resonance as *α* increases above some critical value (in between these for α=2 and α=0.5). For α=0.2 and α=0.5 (middle and right panels) the envelope curve is tangent to the dashed-red at its intersection with the *w*-nullcline, and hence the system exhibits no phase-resonance. In contrast, for α=2 (left panel), the tangent point occurs in between this intersection and the peak of the envelope curve, and thus the system does exhibit phase-resonance.

#### 3.5.3 Negative Values of Both *α* and *ϵ* Amplify the Voltage Response

From (12), negative values of both *α* and *ϵ* reflect the presence of a strong enough amplifying current that makes the effective conductance $g_{L}<0$ and a resonant current ($g_{1}>0$). For the underlying autonomous system to be stable the values of *ϵ* are constrained to be in the region ϵ>−1 (see Fig. 3a). We present a representative example in Fig. 12 (α=−2 and ϵ=−0.5). Comparison with the examples presented in Figs. 10 and 11 demonstrates that the voltage response is more amplified for negative than for positive values of both *α* and *ϵ*. This is true even for negative values of *ϵ* that are not very small in absolute value (time-scale separation no necessarily small).

Geometrically, negative values of *α* cause the slow of the *w*-nullcline to be negative (Fig. 12a) and affect the direction of motion of trajectories. The dynamics of the forced system for very low and very large values of the input frequency *f* is similar to the cases discussed above for positive values of both *α* and *ϵ* (Fig. 12 for f=3 and f=400, respectively). For intermediate values of *f*, the amplification of the voltage response reflects the interaction of the oscillatory inputs with an intrinsic vector field (for the autonomous system) that is correlated with the amplification of the voltage responses to instantaneous inputs discussed in the context of Fig. 4c for negative as compared to positive values of both *α* and *ϵ* (Figs. 4a and 4b).

There are three main differences between the two cases (positive and negative values of both *α* and *ϵ*). First, the phase-resonant frequency $f_{\mathrm{phas}}=138$ is larger than the resonant frequency $f_{\mathrm{res}}=108$ for negative parameter values (Fig. 12b), while $f_{\mathrm{phas}}<f_{\mathrm{res}}$ for positive parameter values. This difference is also captured by the envelope-plane diagram in Fig. 12d. Second, the voltage response is amplified by decreasing, rather than increasing levels of the time-scale separation (given by |ϵ|), for negative parameter values (Figs. 13a and 13b). Finally, the phase profiles are initially at ϕ=−π and increase towards ϕ=π/2 (Fig. 13c) for negative values of both *α* and *ϵ* (compare with Figs. 10 and 11), while *ϕ* never decreases below −π/2 for positive values of these two parameters.

### 3.6 Extension of the Envelope-Plane Diagram Approach to Simple Nonlinear Systems

Linearization around resting potential produces a good approximation to the impedance and phase profiles for weakly forced linear systems. However, significant departures from the linear prediction are expected for larger input amplitudes in the presence of strong nonlinearities, in particular when they are close to the voltage threshold for spike generation. The question arises of how nonlinearities affect the voltage response and, in particular, how the resonant and phase-resonant frequencies depend on the type of these nonlinearities and the time-scale separation between the participating variables.

Linear responses to sinusoidal inputs are characterized by (i) the coincidence of the input and output frequencies, (ii) the proportionality between the output and the input signals that renders the impedance function independent of the input amplitude $A_{\mathrm{in}}$, and (iii) the symmetry between positive and negative values of the voltage response. In terms of the envelope-plane diagrams, the information about the voltage response is captured by the upper envelope curve for $A_{\mathrm{in}}=1$. Here we illustrate how the ideas developed in the previous sections can be extended to the investigation of the mechanisms underlying the generation of resonance and phase-resonance in nonlinear systems.

We use simple piecewise-linear (PWL) systems of the form

(37) $$\frac{dv}{dt}=h_{v}(v)-w+A_{\mathrm{in}}\sin(\Omega t),$$

(38) $$\frac{dw}{dt}=\epsilon \left[h_{w}(v)-w\right],$$

where the functions $h_{v}$ and $h_{w}$ are continuous PWL functions with two linear pieces. We consider two representative cases: (i) $h_{v}$ is PWL and $h_{w}$ is linear, and (ii) $h_{v}$ is linear and $h_{w}$ is PWL. In both cases, the nullclines intersect at the origin. By construction, in a vicinity of the fixed point $\left(\bar{v},\bar{w})=(0,0\right)$ both $h_{v}$ and $h_{w}$ are linear, and system (37)–(38) has the form (14)–(15).

#### 3.6.1 The Voltage Response Is Amplified by Nonlinearities in the Voltage Equation

**Fig. 14:**
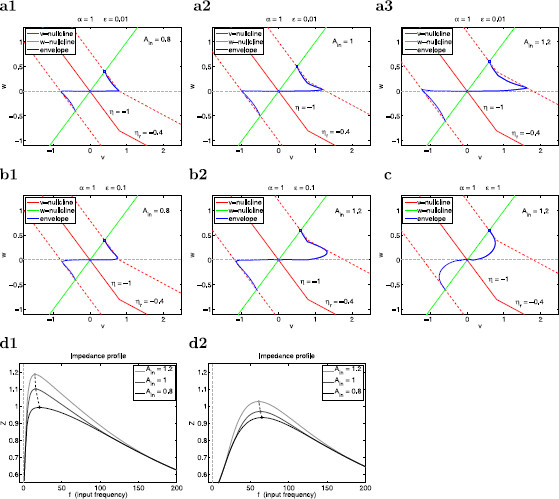
Voltage response of the PWL system (37)–(38) with $h_{v}$ and $h_{w}$ given by (39) to sinusoidal inputs. **a**–**c** Envelope-plane diagrams for representative values of $A_{\mathrm{in}}$ and *ϵ*. **a1**$A_{\mathrm{in}}=0.8$, ϵ=0.01. **a2** $A_{\mathrm{in}}=1$, ϵ=0.01. **a3**$A_{\mathrm{in}}=1.2$, ϵ=0.01. **b1**$A_{\mathrm{in}}=0.8$, ϵ=0.1. **b2**$A_{\mathrm{in}}=1.2$, ϵ=0.1. **c**$A_{\mathrm{in}}=1.2$, ϵ=1. **d** Impedance profiles for representative values of $A_{\mathrm{in}}$ and *ϵ*. **d1**ϵ=0.01. **d2**ϵ=0.1. We used the following parameters: α=1, η=−1, $\eta_{r}=-0.4$, $v_{c}=0.8$

**Fig. 15:**
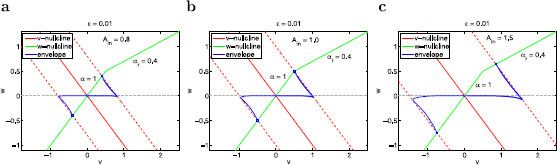
Voltage response of the PWL system (37)–(38) with $h_{v}$ and $h_{w}$ given by (40) to sinusoidal inputs. **a** $A_{\mathrm{in}}=0.8$. **b**$A_{\mathrm{in}}=1$. **c**$A_{\mathrm{in}}=1.5$. We used the following parameters: α=1, η=−1, $\alpha_{r}=0.4$, $v_{c}=0.5$, ϵ=0.01

Here we consider system (37)–(38) with

(39) $$h_{v}(v)=\{\begin{array}{cc} \eta v & \text{if }v\leq 0.8,\\ \eta_{r}v & \text{if }v>0.8 \end{array}\;\text{and}\;h_{w}(v)=\alpha v$$

with η=−1, $\eta_{r}=-0.4$ and α=1. Our results are presented in Fig. 14. Figure 14a shows the envelope-plane diagrams (including both the upper and lower envelopes) for representative values of *α* and *ϵ*. The *v*-nullcline for the unperturbed system (solid-red lines) breaks at v=0.8. For the forced system, the location of the *v*-nullcline changes with *t* and so does the breaking point. The impedance and phase profiles for the parameters in Figs. 15a (ϵ=0.01) and 15b (ϵ=0.1) are presented in Figs. 15d1 and 15d2, respectively. 

For small values of $A_{\mathrm{in}}$ (Figs. 15a1 and 15b1) the dynamics of the PWL system is governed by the underlying linear system and the voltage response is linear. The envelope-plane diagrams are symmetric and similar to the ones discussed in previous sections. For larger values of $A_{\mathrm{in}}$ the voltage response exhibits an amplification that is not captured by the linearization (Figs. 15d1 and 15d2). As $A_{\mathrm{in}}$ increases, the values of both $Z_{\max}$ and $Q_{Z}$ increase, the resonant frequency $f_{\mathrm{res}}$ decreases, and the selectivity increases. Notably, this nonlinear amplification of the voltage response is more pronounced the smaller the value of *ϵ* (larger levels of time-scale separation).

The nonlinear amplification of the voltage response can be understood by comparing the envelope-plane diagrams in Fig. 14a. Geometrically, the nonlinearities are reflected by a sudden change in the slope of the *v*-nullcline. Similarly to the linear case, as *t* progresses, the nonlinear *v*-nullcline moves cyclically between the two dashed-red curves corresponding to $\pm A_{\mathrm{in}}$. The envelope curves live in the region bounded by the *w*-nullcline, the moving *w*-nullcline (dashed-red line) and the *v*-axis (horizontal w=0 line). As $A_{\mathrm{in}}$ increases, the area of this region increases as the triangle in panel a1 becomes a (non-triangular) polygon in Figs. 15a2 and 15a3. These changes are accompanied by an increase in the voltage amplitude of the regions that allow trajectories to reach higher values of *v*.

The nonlinearities do not affect the dynamics of the limit cycle trajectory for low and large values of *f*, which behaves as explained above for linear systems, but they do affect the dynamics of the limit cycle trajectories for intermediate values of *f*. For $A_{\mathrm{in}}=0.8$ (Fig. 15a1), the voltage response is quasi-linear since the nonlinearity is not present in the triangular region where the envelope curve is located. The limit cycle trajectories cannot move beyond the piece of the *v*-nullcline with slope η=−1. For larger values of $A_{\mathrm{in}}$ the nonlinearity moves above the horizontal axis (Figs. 15a2 and 15a3) during the ascending phase of the oscillatory input, thus changing the shape of the envelope-curve region (to a non-triangular polygon).

#### 3.6.2 The Voltage Response Is Amplified by the Time-Scale Separation in the Presence of Nonlinearities in the Voltage Equation

The nonlinear amplification of the voltage response results from the ability of the limit cycle trajectories to move beyond the dashed-red line with slope η=−1. Clearly, this phenomenon is more pronounced for larger values of $A_{\mathrm{in}}$. As expected from our previous discussion for linear systems, this phenomenon is also more pronounced for smaller values of *ϵ* (compare Figs. 14a and 14b) since limit cycle trajectories move in more horizontal directions for smaller values of *ϵ*, and they are able to reach larger voltage values. As *ϵ* increases, limit cycle trajectories move in less horizontal directions and, consequently, they do not take advantage of the extra available region of motion in the envelope-plane created by the nonlinear *v*-nullcline.

We emphasize that the nonlinearities are present, and they are the same, for all values of *ϵ*, but the underlying vector field causes the limit cycle trajectories to “ignore” these nonlinearities and move inside the linear region (Fig. 14d).

The asymmetry in the system’s nonlinearity is reflected in the asymmetry in the voltage responses, which is captured by the differences between the upper and lower envelope curves in the envelope-plane diagrams. Clearly, the difference between these two curves increases as $A_{\mathrm{in}}$ increases.

We note that information about this asymmetric voltage response is not captured by the impedance function.

#### 3.6.3 The Voltage Response Is Almost Insensitive to Nonlinearities in the Gating Variable Equation

Here we consider system (37)–(38) with

(40) $$h_{v}=\eta v\;\text{and}\;h_{w}(v)=\{\begin{array}{cc} \alpha v & \text{if }v\leq 0.5,\\ \alpha_{r}v & \text{if }v>0.5 \end{array}$$

with η=−1, α=1 and $\alpha_{r}=0.4$. Envelope-plane diagrams for representative values of *ϵ* are shown in Fig. 15. The nonlinearities are reflected in the sudden change in the slope of the *w*-nullcline. This change slightly affects the dynamics of the limit cycle trajectories for low values of *f*, since they evolve in close vicinities of the *w*-nullcline but they do not significantly affect the dynamics of the limit cycle trajectory for larger values of *f*. Even for large values of $A_{\mathrm{in}}$ (not shown), the nonlinearities in the *w*-nullcline do not cause any significant change in the envelope-curve region in the envelope-plane, and thus the impedance function does not significantly change with increasing values of $A_{\mathrm{in}}$. The resonant frequency does decrease with increasing values of $A_{\mathrm{in}}$ but significantly less than for the case considered above (not shown).

## 4 Discussion

Subthreshold (membrane potential) resonance has been investigated in a number of neuron types both experimentally and theoretically [10,12-22,29,40], and has been implicated in the generation of preferred neuronal firing rates [11] and network oscillations [23,25,27,41-43]. Phase-resonance has also been investigated in neuronal systems [11,14,29], although to a lesser extent than subthreshold resonance, and it has been proposed to play an important role in neuronal synchronization [30]. 

The voltage response to oscillatory inputs in neurons and other electric circuits is typically characterized in terms of the impedance and phase profiles [10]. For linear systems receiving sinusoidal inputs, these curves can be computed analytically. For nonlinear systems or linear systems receiving non-sinusoidal oscillatory inputs, analytical calculations are not possible, and these curves are computed numerically. 

Even when analytical expressions for the impedance and phase are available, the information they provide about the dynamic mechanisms leading to resonance is limited. For instance, the impedance and phase profiles fail to provide the necessary insight into the roles played by the interaction between the neuron’s intrinsic time scales and the time scales associated with the input currents in the generation of resonance. Furthermore, linearized models fail to capture important nonlinear properties of the voltage response such as its nonlinear amplification for significant levels of the time-scale separation (small values of *ϵ*).

In this paper we have developed and used dynamical system tools to investigate the dynamic mechanisms of generation of subthreshold and phase resonance in two-dimensional linear and linearized (conductance-based) models, and we have extended these tools to include the effect of simple, but not necessarily weak, types of nonlinearities. The mechanistic analysis and the envelope-plane diagrams we developed in this paper provide a framework for the investigation of the preferred frequency responses in three-dimensional and nonlinear models. They can also be used as tools complementary to both the numerically computed and the experimentally measured impedance and phase profiles. More research is needed to adapt these tools to small neuronal networks.

Mechanistic studies of subthreshold resonance have mainly focused on the role that resonant and amplifying currents (and their associated gating variables) play in shaping the neuronal voltage response [10,11,29]. As we have shown in [29], the determination of the attributes and phase profiles as the result of the interaction between these two current types is more complex and not as straightforward as previously thought [10] (see our discussion of resonant and amplifying currents in Sect. 2.5). This is further emphasized by the fact that resonance may occur in the absence of intrinsic oscillations and vice versa [11,29] (see also Fig. 3).

In this study, we set out to understand the dynamic mechanisms underlying the generation of resonance and phase-resonance for a generic class of linearized biophysical models. We were particularly interested in issues that cannot be addressed solely by considering the impedance and phase profiles. These include (i) the identification of the mechanisms of amplification of the voltage response, (ii) the mechanisms of selection of the resonant and phase-resonant frequencies, (iii) the identification of the roles played by the intrinsic time scales and the time scales associated to the current inputs, (iv) the mechanisms that govern their interaction, (v) how all this is affected by changes in the model parameters, (vi) the relation between intrinsic STOs and subthreshold resonance, and (vii) the relation between subthreshold resonance and phase-resonance.

Perhaps our intuition on the resonant properties of neuronal models is based on the dynamics of the so-called *λ*–*ω* systems (55)–(56) (in Appendix A.3), which were used to build the resonate-and-fire models introduced in [31]. These systems display intrinsic oscillations with natural frequency $\Omega_{\mathrm{nat}}=\omega$ for all values of *λ* (58). They also exhibit resonance and phase-resonance. The natural, resonant and phase-resonant frequencies (59) coincide for λ=0 and they are different for other values of *λ*. Although *λ*–*ω* systems have been used to investigate the dynamics of resonant neurons [31], they are not representative of linearized neuronal models [11]. They rather correspond to cases where the voltage and gating variables evolve with comparable rates (ϵ=1) (see (61)–(62) in Appendix A.3), and leave out the large class of neuron types that have a strong time-scale separation (*ϵ* is small) in the subthreshold regime.

The fact that resonance may occur in the absence of intrinsic STOs for non-negligible parameter regimes [11,29] implies that resonance is not necessarily reflecting the amplification of an existing intrinsic oscillation by an oscillatory input current. Instead, resonance is uncovering the ability of the neuron to operate at time scales that are neither intrinsic nor imposed by the oscillatory input, but they emerge as the result of the interaction between a neuron’s intrinsic time scale (determined by the neuron’s intrinsic properties) and the time scales of the oscillatory input. These emergent time scales are likely to be the ones that play a significant role in network interactions. 

To address these mechanistic issues from a geometric/dynamic perspective, we have extended the classical phase-plane analysis approach to include the effects of oscillatory inputs. This approach consists of projecting the three-dimensional space (for *v*, *w* and *t*) onto the two-dimensional plane (for *v* and *w*) and viewing the projection of the two-dimensional *v*-nullsurface (spanned by the *v*-nullcline for the autonomous system and *t*) as a moving one-dimensional *v*-nullcline as *t* progresses. Trajectories track the motion of the *v*-nullcline and the fixed point with a speed that depends on the underlying vector field and the input frequency *f*. The shapes of the limit cycle trajectories depend on *f*. A system exhibits resonance if for some value of *f* the amplitude of the limit cycle trajectory in the *v*-direction is larger than for f=0. Qualitative predictions on the effect of changes in parameters on both resonance and phase-resonance can be made by looking at the phase-plane and the associated envelope-plane diagrams. Approaches including “moving nullclines” have been used before to investigate the mechanisms of synchronization of neuronal [44-47] and other systems [48,49] but they have not been used before in the context of the analysis of subthreshold and phase resonance. 

From a geometric perspective, we view both resonance and phase-resonance as the result of the interaction between the time scales of the neuron (determined by the geometry of the unperturbed phase plane and the intrinsic time-scale separation) and the oscillatory input (captured by the moving *v*-nullcline). The resonant frequency, if it exists, is the input frequency at which the voltage responds optimally to the oscillatory driving current, which is captured by the cyclic movement of the *v*-nullcline. The trajectory’s response is neither too fast nor too slow so that the voltage is able to reach a higher value than for other input frequencies. The time scale associated to the zero-phase frequency is also an emergent time scale, and is optimal in the sense that the trajectory is neither too fast nor too slow so that the trajectory intersects the moving *v*-nullcline at the exact time at which the latter reaches its maximum; i.e., both the voltage response and the input current peak at the same time. We showed that these two phenomena are captured by the shape of the envelope-plane diagrams not only for linear models, but also for nonlinear ones.

The concept of time scale for neural models is not always easy to quantify. In some limiting cases the time constants provide reliable information about the rate of change of the participating variables. This is the case for the one-dimensional passive membrane equation and for the so-called slow–fast systems [47]. However, time constants do not always capture the effective time scales. For the purpose of our study, we have qualitatively characterized the effective time scales of the isolated neurons by looking at their response to instantaneous DC (tonic) inputs and the shapes of the corresponding trajectories. We have identified the effective time scales of the isolated neurons that are relevant for their interaction with oscillatory inputs with the time scales that govern the behavior of the initial portion of the trajectory from its initial point until it reaches its highest voltage value. Geometrically, this is determined by the crossing point with the *v*-nullcline displaced by $A_{\mathrm{in}}$ units (dotted-red *v*-nullclines). The effective time scales defined in this way may or may not represent the dynamic behavior of the autonomous trajectories for all subsequent times *t*, depending on the parameter values, but they are appropriate to describe the interaction between neurons and oscillatory inputs. As we showed, trajectories reverse direction after crossing the moving *v*-nullclines.

This notion of effective time scales for the underlying autonomous system is connected with its eigenvalues. However, our discussion of resonance is independent of the behavior of the autonomous system for large values of *t*. Therefore, the mechanisms of generation of resonance are in general independent from those responsible for the generation of intrinsic STOs.

The mechanism of generation of intrinsic STOs in linear systems is well understood [50]. The natural frequency ($\Omega_{\mathrm{nat}}$) corresponds to the eigenvalues’ imaginary part. The eigenvalues’ real part affects the amplitude of solutions, and consequently the evolution of trajectories in the phase plane, without affecting the oscillation frequency. The initial time interval of transient behavior that determines the effective time scales depends on both the eigenvalues’ real part and $\Omega_{\mathrm{nat}}$, and not only on $\Omega_{\mathrm{nat}}$. Only when the effect of the eigenvalues’ real part is small enough, both the generation of intrinsic STOs and resonance are governed by a similar underlying mechanism as occurs for the *λ*–*ω* systems discussed above.

To our knowledge, no previous study has addressed questions on subthreshold resonance in nonlinear systems from an analytical perspective. In this paper, we have extended our analysis for linear systems to include simple, piecewise-linear types of nonlinearities in both the equations for *v* and *w*. Our main question was: to what extent does the linear prediction capture the nonlinear effects? We found that when the *v*-nullcline is nonlinear the differences between the nonlinear response and the linear prediction increase not only with increasing values of the input amplitude $A_{\mathrm{in}}$ but also with increasing levels of the time-scale separation between the voltage and the gating variable (decreasing values of *ϵ*). These differences almost disappear when both equations evolve at comparable rates. In contrast, voltage responses are almost insensitive to nonlinearities located in the gating variable equation. In these two latter cases, the nonlinearities are present in the system but the voltage response does not detect them. More research is needed to understand whether these findings play out in more realistic nonlinear models.

Resonance has been first studied in the damped harmonic oscillator subject to sinusoidal forcing (see Appendix A.4). Similar to the *λ*–*ω* system, the natural and resonant frequencies coincide when the system is undamped and they are different in the damped case. Unlike the neural models discussed in this paper, the over-damped system (real eigenvalues) does not exhibit resonance. The damped harmonic oscillator can be rewritten as a system of two first order equations (64)–(65). However, differently from the systems considered in this paper, the sinusoidal forcing is located in the second equation rather than the first. In other words, the forcing term does not directly affect the dynamics of the variable for which we compute the response to the oscillatory input (analogous to *v*), but its derivative (analogous to *w*). Therefore, one should not necessarily expect resonance to occur in the absence of intrinsic oscillations. The geometric ideas developed in this paper can adapted to these systems. The moving nullcline will be the analog to the *w*-nullcline (oblique with negative slope) instead of the *v*-nullcline. The latter will be horizontal and fixed.

## Appendix A: Forced Two-dimensional Linear Systems: Eigenvalues, Natural Frequency, and Impedance and Phase Profiles

We consider the following forced two-dimensional linear system:

(41) $$\frac{dx}{dt}=ax+by+A_{\mathrm{in}}\sin(\Omega t),$$

(42) $$\frac{dy}{dt}=cx+dy,$$

where *a*, *b*, *c*, and *d* are constant, Ω>0 and $A_{\mathrm{in}}\geq 0$.

### A.1 Intrinsic Oscillations and Natural Frequency

The Jacobian matrix for the corresponding autonomous system ($A_{\mathrm{in}}=0$) is given by

(43) $$J=(\begin{array}{cc} a & b\\ c & d \end{array}).$$

The roots of the characteristic polynomial are given by

(44) $$r_{1,2}=\frac{(a+d)\pm \sqrt{{\left(a-d\right)}^{2}+4bc}}{2}.$$

From (44), the unforced system displays oscillations for values of the parameters satisfying

(45) $$4bc+{\left(a-d\right)}^{2}<0,$$

with the natural frequency given by

(46) $$\Omega_{\mathrm{nat}}=\frac{\sqrt{-4bc-{\left(a-d\right)}^{2}}}{2}.$$

### A.2 Voltage Response to Sinusoidal Inputs: Impedance Amplitude and Phase

System (41)–(42) can be written as a second order linear ODE,

(47) $$\frac{d^{2}x}{dt^{2}}-(a+d)\frac{dx}{dt}+(ad-bc)x=A_{\mathrm{in}}\left[-d\sin(\Omega t)+\Omega \cos(\Omega t)\right].$$

The particular solution to system (47) has the form

(48) $$x_{p}(t)=A_{\mathrm{out},1}\sin(\Omega t)+A_{\mathrm{out},2}\cos(\Omega t)$$

with

(49) $$A_{\mathrm{out},1}=-\frac{(ad-bc-\Omega^{2})d+(a+d)\Omega^{2}}{{\left[ad-bc-\Omega^{2}\right]}^{2}+{\left(a+d\right)}^{2}\Omega^{2}}A_{\mathrm{in}}$$

and

(50) $$A_{\mathrm{out},2}=\frac{(ad-bc-\Omega^{2})\Omega -(a+d)\Omega d}{{\left[ad-bc-\Omega^{2}\right]}^{2}+{\left(a+d\right)}^{2}\Omega^{2}}A_{\mathrm{in}}.$$

The solution (48) can be written as

(51) $$x_{p}(t)=A_{\mathrm{out}}\sin(\Omega t-\phi ).$$

The impedance amplitude and phase are given by (see Appendix A.5)

(52) $$Z^{2}(\Omega ):=\frac{A_{\mathrm{out}}^{2}}{A_{\mathrm{in}}^{2}}=\frac{A_{\mathrm{out},1}^{2}+A_{\mathrm{out},2}^{2}}{A_{\mathrm{in}}^{2}}=\frac{d^{2}+\Omega^{2}}{{\left[ad-bc-\Omega^{2}\right]}^{2}+{\left(a+d\right)}^{2}\Omega^{2}}$$

and

(53) $$\phi (\Omega )=-\tan^{-1}\left(\frac{A_{\mathrm{out},2}}{A_{\mathrm{out},1}}\right)=\tan^{-1}\frac{(ad-bc-\Omega^{2})\Omega -(a+d)\Omega d}{(ad-bc-\Omega^{2})d+(a+d)\Omega^{2}},$$

respectively. The impedance *Z* peaks at the resonant frequency

(54) $$\Omega_{\mathrm{res}}=\sqrt{-d^{2}+\sqrt{b^{2}c^{2}-2abcd-2d^{2}bc}},$$

provided the quantity inside the radical is positive.

### A.3 The *λ*–*ω* Systems with Sinusoidal Forcing

The so-called *λ*–*ω* systems [51] with sinusoidal forcing have the form

(55) $$\frac{dx}{dt}=-\lambda x-\omega y+B_{\mathrm{in}}\sin(\Omega t),$$

(56) $$\frac{dy}{dt}=\omega x-\lambda y,$$

with λ>0, ω>0 and $B_{\mathrm{in}}\geq 0$. System (55)–(56) can be written as the following second order linear ODE:

(57) $$\frac{d^{2}x}{dt^{2}}+2\lambda \frac{dx}{dt}+\left(\lambda^{2}+\omega^{2}\right)x=B_{\mathrm{in}}\left[\lambda \sin(\Omega t)+\Omega \cos(\Omega t)\right].$$

The eigenvalues and natural frequency of the autonomous system are given by (see Appendix A.1)

(58) $$r_{1,2}=-\lambda \pm \sqrt{-\omega^{2}}\;\text{and}\;\Omega_{\mathrm{nat}}=\omega .$$

The resonant and phase-resonant frequencies are given by (see Appendix A.2)

(59) $$\Omega_{\mathrm{res}}=\sqrt{-\lambda^{2}+\omega \sqrt{4\lambda^{2}+\omega^{2}}}\;\text{and}\;\Omega_{\mathrm{phas}}=\sqrt{\omega^{2}-\lambda^{2}},$$

provided the quantities inside the radicals are positive. For λ=0,

(60) $$\Omega_{\mathrm{nat}}=\Omega_{\mathrm{res}}=\Omega_{\mathrm{phas}}.$$

System (55)–(56) can be transformed into a system of the form (14)–(15) by defining

(61) $$v=\lambda x,\;\;w=\omega y,\;\;\hat{t}=\lambda t,$$

and

(62) $$\alpha =\frac{\omega^{2}}{\lambda^{2}},\;\;\epsilon =1.$$

### A.4 The Harmonic Oscillator with Sinusoidal Forcing

The equation for the harmonic oscillator with sinusoidal forcing reads

(63) $$\frac{d^{2}x}{dt^{2}}+\beta \frac{dx}{dt}+\gamma x=C_{\mathrm{in}}\sin(\Omega t),$$

where β≥0 and γ>0. By defining y=−dx/dt, (63) can be rewritten as the following two-dimensional linear system of ODEs:

(64) $$\frac{dx}{dt}=-y,$$

(65) $$\frac{dy}{dt}=\gamma x-\beta y-C_{\mathrm{in}}\sin(\Omega t).$$

Differently from the systems considered in this paper, and the general form (41)–(42), the sinusoidal forcing is located in the second equation rather than the first. We note that the two equations are not interchangeable since we are following the convention that the first equation describes the dynamics of the variable (*x*) for which we compute the response to the oscillatory input.

The eigenvalues of the autonomous system are given by

(66) $$r_{1,2}=\frac{-\beta \pm \sqrt{\beta^{2}-4\gamma }}{2}.$$

The natural frequency of the autonomous system is given by

(67) $$\Omega_{\mathrm{nat}}=\frac{\sqrt{4\gamma -\beta^{2}}}{2}=\sqrt{\gamma -\frac{\beta^{2}}{4}},$$

provided $4\gamma -\beta^{2}>0$. The impedance is given by

(68) $$Z(\Omega )=\frac{1}{{\left(\gamma -\Omega^{2}\right)}^{2}+\beta^{2}\Omega^{2}}.$$

Note that the formula (54) is not applicable in this case and the impedance has to be calculated separately. The impedance peaks at

(69) $$\Omega_{\mathrm{res}}=\sqrt{\gamma -\frac{\beta^{2}}{2}},$$

provided the quantity inside the radical is positive. For β=0, $\Omega_{\mathrm{nat}}=\Omega_{\mathrm{res}}=\sqrt{\gamma }$. For other values of *β* for which both $\Omega_{\mathrm{nat}}$ and $\Omega_{\mathrm{res}}$ are defined, $\Omega_{\mathrm{nat}}\neq \Omega_{\mathrm{res}}$. If the eigenvalues are real, the over-damped harmonic oscillator does not exhibit resonance since the inequalities $4\gamma -\beta^{2}<0$ and $2\gamma -\beta^{2}>0$ cannot be simultaneously satisfied.

System (64)–(65) can be rescaled by defining

(70) $$\hat{t}=\beta t\;\text{and}\;\hat{y}=\frac{\beta }{\gamma }y$$

and substituting into system (64)–(65). The resulting equations read

(71) $$\frac{dx}{d\hat{t}}=-\frac{\gamma }{\beta^{2}}\hat{y},$$

(72) $$\frac{d\hat{y}}{d\hat{t}}=x-\hat{y}-\frac{C_{\mathrm{in}}}{\gamma }\sin(\Omega \hat{t}/\beta ).$$

### A.5 Oscillatory Inputs: Additional Calculations

For a sinusoidal input of the form $F(t)=A_{\mathrm{in}}\sin(\Omega t)$ the system’s output will be a function

(73) $$X(t)=A_{\mathrm{out}}\sin(\Omega t-\phi ).$$

Equation (73) can be rewritten as follows:

(74) $$X(t)=A_{\mathrm{out}}\cos\phi \sin(\Omega t)-A_{\mathrm{out}}\sin\phi \cos(\Omega t)$$

or

(75) $$X(t)=A_{\mathrm{out},1}\sin(\Omega t)+A_{\mathrm{out},2}\cos(\Omega t)$$

with

(76) $$A_{\mathrm{out},1}=A_{\mathrm{out}}\cos\phi ,\;\;A_{\mathrm{out},2}=-A_{\mathrm{out}}\sin\phi .$$

Solving for $A_{\mathrm{out}}$ and *ϕ* we obtain

(77) $$A_{\mathrm{out}}^{2}=A_{\mathrm{out},1}^{2}+A_{\mathrm{out},2}^{2}$$

and

(78) $$\phi =-\tan^{-1}\left(\frac{A_{\mathrm{out},2}}{A_{\mathrm{out},1}}\right).$$

From (77)

(79) $$Z^{2}(\Omega )=\frac{A_{\mathrm{out},1}^{2}+A_{\mathrm{out},2}^{2}}{A_{\mathrm{in}}^{2}}.$$

## Appendix B: Biophysical $I_{h}+I_{\mathrm{Nap}}$ and $I_{\mathrm{Ks}}+I_{\mathrm{Nap}}$ Models

### B.6 $I_{h}+I_{\mathrm{Nap}}$ Model

This model has been proposed in [37]. It has a persistent sodium current and a two-component (fast and slow) h-current given by $I_{p}=G_{p}p(V-E_{\mathrm{Na}})=G_{p}p_{\infty }(V)(V-E_{\mathrm{Na}})$ and $I_{h}=G_{h}r(V-E_{h})=G_{h}(c_{f}r_{f}+c_{s}r_{s})(V-E_{h})$, respectively, with $E_{h}=-20\text{ mV}$, $E_{\mathrm{Na}}=55\text{ mV}$, $c_{f}=0.65$, and $c_{s}=0.35$. The voltage-dependent activation/inactivation and time constants are given by

$$\begin{array}{rcl} p_{\infty }(V) & = & 1/\left(1+e^{-(V+38)/6.5}\right),\\ \tau_{p}(V) & = & 0.15,\\ r_{f,\infty }(V) & = & 1/\left(1+e^{(V+79.2)/9.78}\right),\\ \tau_{r_{f}}(V) & = & 0.51/\left(e^{(V-1.7)/10}+e^{-(V+340)/52}\right)+1,\\ r_{s,\infty } & = & 1/\left(1+e^{(V+71.3)/7.9}\right),\\ \tau_{r_{s}}(V) & = & 5.6/\left(e^{(V-1.7)/14}+e^{-(V+260)/43}\right)+1. \end{array}$$

For the two-dimensional model used in this paper, the $c_{f}=1$ and $c_{s}=0$.

### B.7 $I_{\mathrm{Ks}}+I_{\mathrm{Nap}}$ Model

This model has been proposed in [37]. It has a persistent sodium current and a slow potassium (M-type) current given by $I_{p}=G_{p}p(V-E_{\mathrm{Na}})=G_{p}p_{\infty }(V)(V-E_{\mathrm{Na}})$ and $I_{\mathrm{Ks}}=G_{q}q(V-E_{x_{k}})$ with $E_{\mathrm{Na}}=55\text{ mV}$ and $E_{k}=-90\text{ mV}$. The voltage-dependent activation/inactivation and time constants are given by

$$\begin{array}{rcl} p_{\infty }(V) & = & 1/\left(1+e^{-(V+38)/6.5}\right),\\ \tau_{p}(V) & = & 0.15,\\ q_{\infty }(V) & = & 1/\left(1+e^{-(V+35)/6.5}\right),\\ q_{\tau }(V) & = & 90. \end{array}$$

## Competing Interests

The author declares that he has no competing interests.
